# A Chromosome-Level Assembly of Blunt Snout Bream (*Megalobrama amblycephala*) Genome Reveals an Expansion of Olfactory Receptor Genes in Freshwater Fish

**DOI:** 10.1093/molbev/msab152

**Published:** 2021-05-18

**Authors:** Han Liu, Chunhai Chen, Maolin Lv, Ning Liu, Yafei Hu, Hailin Zhang, Erik D Enbody, Zexia Gao, Leif Andersson, Weimin Wang

**Affiliations:** 1 College of Fisheries, Key Lab of Agricultural Animal Genetics, Breeding and Reproduction of Ministry of Education/Key Lab of Freshwater Animal Breeding, Ministry of Agriculture and Rural Affairs, Huazhong Agricultural University, Wuhan, China; 2 Hubei Hongshan Laboratory, Engineering Research Center of Green Development for Conventional Aquatic Biological Industry in the Yangtze River Economic Belt, Ministry of Education, Wuhan, China; 3 BGI Genomics, BGI-Shenzhen, Shenzhen, China; 4 Science for Life Laboratory, Department of Medical Biochemistry and Microbiology, Uppsala University, Uppsala, Sweden; 5 Department of Veterinary Integrative Biosciences, Texas A&M University, College Station, TX, USA; 6 Department of Animal Breeding and Genetics, Swedish University of Agricultural Sciences, Uppsala, Sweden

**Keywords:** Cyprinid fish, comparative genomics, olfactory receptor, evolutionary dynamics, ecological adaptation

## Abstract

The number of olfactory receptor genes *(ORs*), which are responsible for detecting diverse odor molecules varies extensively among mammals as a result of frequent gene gains and losses that contribute to olfactory specialization. However, how OR expansions/contractions in fish are influenced by habitat and feeding habit and which OR subfamilies are important in each ecological niche is unknown. Here, we report a major *OR* expansion in a freshwater herbivorous fish, *Megalobrama amblycephala*, using a highly contiguous, chromosome-level assembly. We evaluate the possible contribution of *OR* expansion to habitat and feeding specialization by comparing the OR repertoire in 28 phylogenetically and ecologically diverse teleosts. In total, we analyzed > 4,000 *ORs* including 3,253 intact, 122 truncated, and 913 pseudogenes. The number of intact *ORs* is highly variable ranging from 20 to 279. We estimate that the most recent common ancestor of Osteichthyes had 62 intact *ORs*, which declined in most lineages except the freshwater Otophysa clade that has a substantial expansion in subfamily β and ε *ORs*. Across teleosts, we found a strong association between duplications of β and ε ORs and freshwater habitat. Nearly, all *ORs* were expressed in the olfactory epithelium (OE) in three tested fish species. Specifically, all the expanded β and ε *ORs* were highly expressed in OE of *M. amblycephala*. Together, we provide molecular and functional evidence for how OR repertoires in fish have undergone gain and loss with respect to ecological factors and highlight the role of β and ε *OR* in freshwater adaptation.

## Introduction

Olfaction is an important physiological function in animals because of its role in foraging, mate selection and avoiding predators or poisonous agents (Hara 1975; Su et al. 2009; Bazáes et al. 2013; Hughes et al. 2018). The vertebrate olfactory system is able to detect and discriminate various odor molecules in the environment using the multigene family of olfactory receptor genes (*ORs*). Vertebrate ORs belong to the family of G-protein-coupled receptors that are composed of seven α-helical transmembrane (TM) regions with conserved motifs (Mombaerts 1999). *OR* genes are predominantly expressed in sensory neurons of the main olfactory epithelium (OE) in nasal cavities both in mammals (Vassar et al. 1993; van der Linden et al. 2018) and fish (Churcher et al. 2015; Cong et al. 2019). The diversity and large number of *OR* genes facilitate the discrimination of a diverse range of environmental odor particles and are thought to be critical for adaptation to local environmental conditions.

The number of intact *OR* genes varies extensively among species of placental mammals ranging from ∼300 in orangutan to ∼2,000 in African elephants, and most species have a substantial number of *OR* pseudogenes (Niimura et al. 2014). The dramatic differences in OR repertoire and gene numbers among vertebrates result from frequent gene gains and losses through duplication and pseudogenization during evolution (Vandewege et al. 2016; Hughes et al. 2018; Niimura et al. 2018). As a consequence, such dynamic evolution of OR repertoire likely facilitates adaptation to different ecological niches (e.g., feeding ecology and habitat) (Hayden et al. 2010, 2014; Vandewege et al. 2016; Hughes et al. 2018). In mammals, aquatic and terrestrial species differ in total number of *OR* genes per gene family (Hayden et al. 2010, 2014) and the OR repertoire has not only expanded but also contracted in association with changes to local environmental conditions (Hughes et al. 2018). Together, these results suggest that *OR* gene expansion has played an important role in ecological adaptation in mammals, but comparatively less is known about the role of water-soluble *OR* in ecological adaptation in fish.

*OR* genes in vertebrates are classified into nine subfamilies, α, β, γ, δ, ε, ζ, η, θ, and κ (Niimura and Nei 2005; Niimura 2009). Most mammalian *OR* genes belonging to subfamily α and λ, known as “mammalian-like” genes, are expressed in air-filled medial diverticulum and responsible for detecting airborne odorants, whereas fish express subfamily δ, ε, ζ, and η, referred to as “fish-like” genes, are expressed in water-filled lateral diverticulum and associated with detecting water-soluble odorants. These water-soluble odorants mainly include amino acids, bile acids, gonadal steroids, and prostaglandins, which are nonvolatile (Hara and Zhang 1996; Cong et al. 2019). Because subfamily β *OR* genes were present both in aquatic and terrestrial vertebrates, they are recognized as both water soluble and airborne odorants (Niimura 2009).

Fish represent one of the largest vertebrate groups with at least 20,000 known species, colonizing tropical, temperate, and polar waters as well as virtually all fresh-water environments. Like other vertebrates, the fish olfactory system is critical for behavior related to feeding, reproduction, predator avoidance and odorant-oriented migration (Laberge and Hara 2001; Hamdani and Døving 2007). The recent availability of a wide taxonomic breadth of fish genome sequences offers an opportunity to explore the evolution of OR repertoires in this group. To date, most previous fish OR studies only focused on the gene identification, whereas the evolutionary dynamics of fish *OR* gene families and their role in adaptation to different ecological niches are completely unknown.

We tested the hypothesis that the evolution of fish *OR* gene repertoire has been influenced by habitat and feeding habit and identified which *OR* gene subfamilies are important for each ecological niche. We generated a high-quality reference genome assembly of a commercially important herbivorous fish, the blunt snout bream (*Megalobrama amblycephala*), to explore the *OR* gene family repertoire across 28 phylogenetic and ecologically diverse fish species.

## Results

### Genome Sequencing Assembly and Annotation

**Fig. 1.**
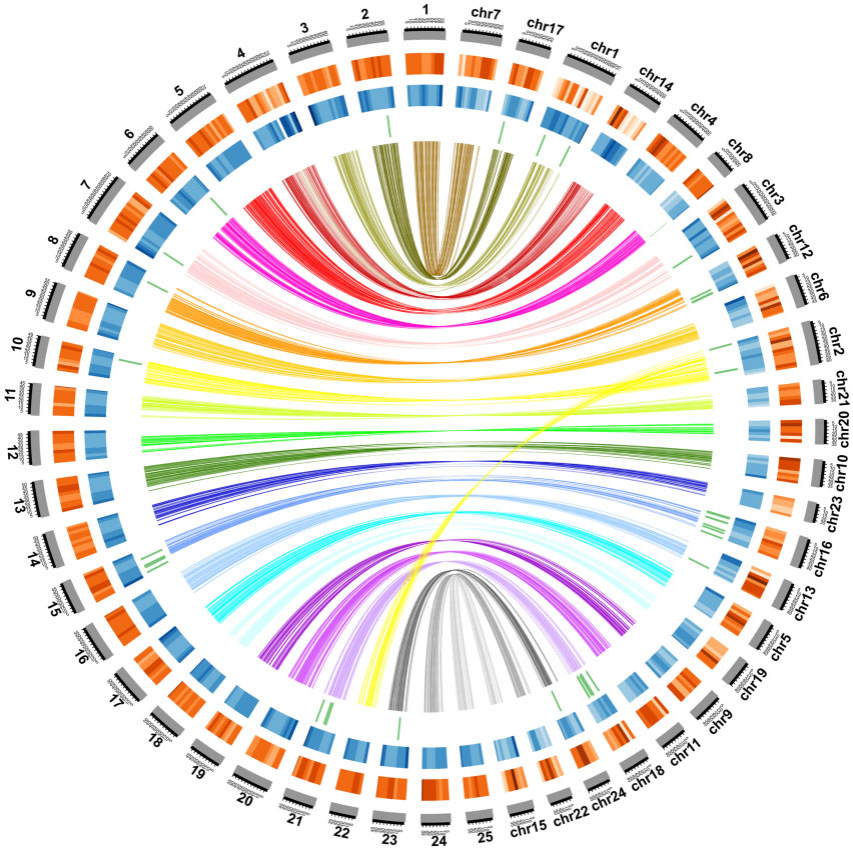
Whole genome alignment between *M. amblycephala* (right) and *D. rerio* (left). From inside to outside: green bars, OR genes; blue bars, GC content within a 50-kb sliding window; orange bars, gene distribution on each chromosome; gray, the genetic linkage map.

We produced a high quality reference genome using a combination of PacBio long reads (supplementary table 1, Supplementary Material online) and Illumina mate pair and paired end sequencing. The assembled genome includes 1,522 contigs and 868 scaffolds with a total length of 1.11 Gb (supplementary table 2, Supplementary Material online). BUSCO analysis identified 96.1% complete and 1.3% partial genes from the 4,584-gene Actinopterygii reference data set (supplementary table 3, Supplementary Material online). We used our previously published high-density linkage map (Liu et al. 2017) to order scaffolds and generate a chromosome-level assembly. Of 868 scaffolds in the assembly, 650 anchored onto 24 chromosomes with a total length of 1,078 Mb, representing 97.2% of the assembled genome sequences (table 1; supplementary table 4, Supplementary Material online). Although *M. amblycephala* shared a most recent common ancestor (MRCA) with *Danio rerio* ∼54 Ma (Liu et al. 2017), the genome of *M. amblycephala* retained strong collinearity with that of *D. rerio* (fig. 1). No large interchromosomal translocation was found between the 25 chromosomes of *D. rerio* and the 24 chromosomes of *M. amblycephala*. One notable difference is that *M. amblycephala* Chr2 corresponds to *D. rerio* Chr10 and 22 (fig. 1). We used Maker (Holt and Yandell 2011) to annotate the novel reference genome and quantify *OR* genes copies in *M. amblycephala*. In doing so, we discovered a notable expansion of *OR* genes that are mainly located on Chr16 and 18 (fig. 1). To evaluate whether this expansion was associated with ecological factors, we performed comparative analysis across other fish species with variation in habitat.

**Table 1:** Summary statistics for different assemblies of the *M. amblycephala* genome

	V1.0	V2.0
Total genome size (Mb)	1,116	1,109
N50 length of scaffold (kb)	840	3,154
N50 length of contig (kb)	49	2,397
Total GC content (%)	37.30	37.64
Complete BUSCOs (%)	81.4	96.1
Fragmented BUSCOs (%)	9.1	1.3
Protein-coding genes number	23,696	30,357
Average gene length (bp)	15,797	16,093
Number of markers in genetic map	5,317	13,653
Scaffolds anchored on linkage groups (LGs)	1,434	650
Length of scaffolds anchored on LGs (Mb)	779.54 (70.0%)	1,078 (97.2%)

V1.0 is the version published in Liu et al. (2017); V2.0 is the final hybrid assembly in this study.

### Identification of or Repertoires Across Fish Genomes

**Fig. 2.**
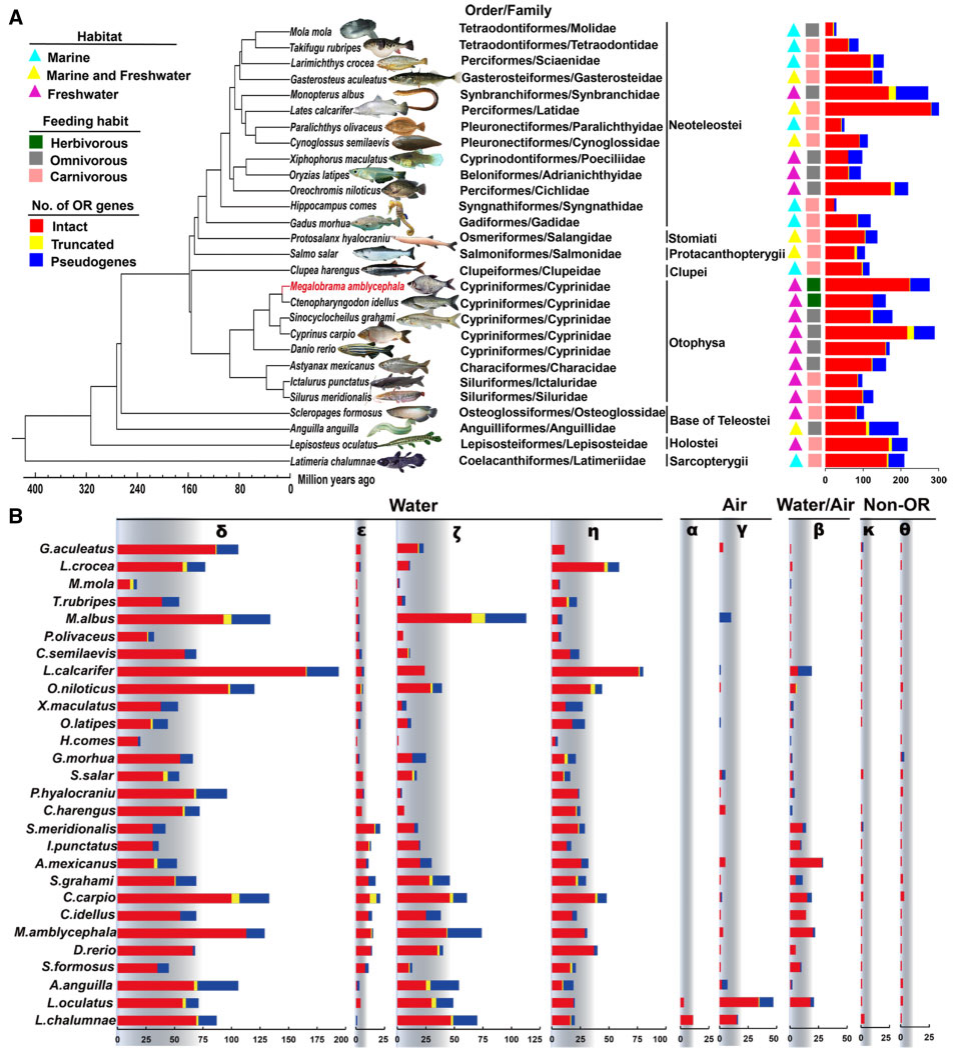
Phylogenetic tree and number of *OR* genes in 28 fish species. (*A*) Phylogenetic tree depicting the evolutionary relationships of 28 fish species. Genome of *M. amblycephala* sequenced in this study is highlighted in red. Habitat and feeding habits are depicted for each species. Bars represent the total *OR* numbers. (*B*) The number of *OR* genes from each subfamily across 28 fish species. Red, yellow and blue bars represent intact genes, truncated genes and pseudogenes, respectively. “Water,” “Air,” and “Water/Air” represent the detection of water-soluble, airborne and both water and airborne odorants, respectively.

We identified the olfactory genes in the genome assemblies of *M. amblycephala* and 27 other fish species for which deep-coverage genome sequences are available (supplementary table 5, Supplementary Material online). These 28 species span 19 different fish orders and include seven other Otophysa species in addition to *M. amblycephala* (fig. 2*A*). Following an extensive homology search and manual curation, we identified 4,288 *OR* genes and classified them into three categories, intact genes (putatively functional genes, *n* = 3,253), truncated genes (*n* = 122), and pseudogenes (*n* = 913, fig. 2*A*). The proportion of pseudogenes among fish species was highly variable and ranged from 5.3% in *D. rerio* to 37.8% in *Xiphophorus maculatus*. We also found extensive variation in the size of OR repertoires and the number of intact genes ranged from 20 in *Mola* to 279 in *Lates calcarifer* while the reference, *M. amblycephala*, contained 223 intact *OR* genes (fig. 2*A*). The number of *OR* genes varies in a lineage-specific manner, for example, as in Tetraodontiformes, *M. mola* and *Takifugu rubripes* which have low numbers of *OR* (20 and 61, respectively) whereas in Cypriniformes we found more than 120 *OR* genes in each species.

### OR Gene Family Phylogeny and Classification

To examine the evolutionary relationships and classify the subfamilies of *OR* genes, we constructed a Maximum Likelihood (ML) tree using 3,253 amino acid sequences from 28 fish genomes (supplementary fig. 1, Supplementary Material online). Here, we restricted our analysis to only include intact genes because most pseudogenes contained deletions and truncated genes were much shorter than the intact genes. The *OR* genes clearly separated into nine a priori subfamilies α, β, γ, δ, ε, ζ, η, θ, and κ (Niimura 2009) and the major clades of the tree were supported by high bootstrap values (more than 70% in 1,000 replicates). The δ subfamily is the largest subfamily accounting for more than 50% of the total number of identified *ORs* followed by subfamily ζ and η. *OR* genes generally form tandem arrays that are highly conserved across distantly related species (supplementary fig. 2, Supplementary Material online).

The number of genes belonging to *OR* subfamilies is highly variable among fish species (fig. 2*B*;supplementary table 6, Supplementary Material online). *OR* genes belonging to subfamily α are almost completely absent in fish with the exception of 3 and 11 copies in *Lepisosteus oculatus* and *Latimeria chalumnae*. Interestingly, the airborne odorants subfamily γ of *OR* genes, is present in low numbers with one to five copies in some teleost fish (*Gasterosteus aculeatus*, *Oreochromis niloticus*, *Salmo salar*, *Protosalanx hyalocranius*, *Clupea harengus*, *Astyanax mexicanus*, *Scleropages formosus*, *Anguilla*, and five Cyprinid fishes) but with 34 copies in *L. oculatus* and 15 in *L. chalumnae* (fig. 2*B*;supplementary table 6, Supplementary Material online). Subfamily δ, ε, ζ, η, and β *OR* genes are abundant in all tested fish (supplementary table 6, Supplementary Material online). Particularly notable expansions include β and ε *OR* genes, which have expanded in eight Otophysa fish species with 5 − 28 and 9 − 16 copies, respectively (fig. 2*B*;supplementary table 6, Supplementary Material online).

### Gains and Losses of OR Genes in Fish during Evolution

**Fig. 3.**
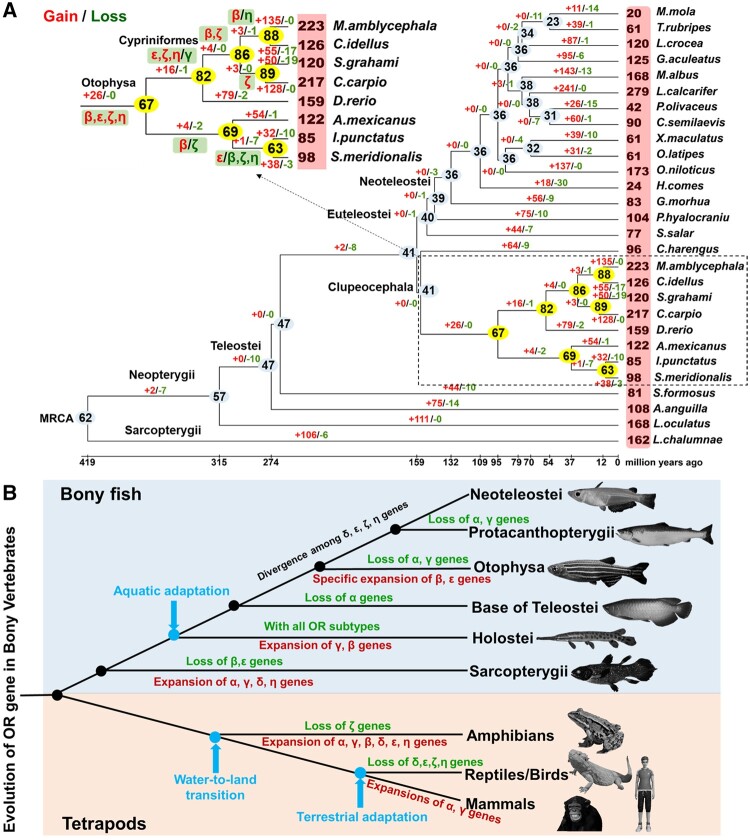
Evolutionary dynamics of OR genes in fish and tetrapod. (*A*) Changes in the number of OR genes during the evolution of 28 fish species inferred from intact genes. Numbers in the red rectangular box indicates the number of intact OR genes in each fish species. The estimated number in light blue and yellow oval represents the number of OR genes at an ancestral node. Estimated numbers of gene gains and gene losses in each branch are also shown with red plus and green minus signs. (*B*) Schematic illustration of the evolution of OR gene families in bony vertebrates.

To investigate the evolutionary dynamics in the number of *OR* genes in teleost, we estimated the OR repertoire size in ancestral species and calculated the numbers of gene gains and losses for each branch during the evolution and speciation of the 28 fish species. The results showed that gains and losses of *OR* genes have occurred frequently in each taxonomic lineage (fig. 3*A*). Two species with similar numbers of *OR* genes may have very different *OR* gene repertoires. For example, *X. maculatus* and *Oryzias latipes* both have 61 *OR* genes at present, whereas their common ancestor was estimated to have 32 (fig. 3*A*). Similarly, in *Silurus meridionalis* and *Ictalurus punctatus*, each species has ∼90 *ORs*, but only ∼60 is shared between them. The MRCA of Osteichthyes was estimated to have had 62 intact ancestral *OR* genes with all subfamilies represented. Several major gain and loss events happened, including the loss of 10 *OR* genes in the Teleostei lineage and 26 gained in the Otophysa clade when compared with their MRCA, Neopterygii and Clupeocephala lineage, respectively. These 26 gained *OR* genes belong to subfamily β, ε, ζ, and η (fig. 3*A*).

In mammals and birds, the number of subfamily α and γ subfamilies of *OR* genes are preferentially expanded (fig. 3*B*), whereas subfamily δ, ε, ζ, and η were completely lost (Niimura 2009). Amphibians retain nearly all *OR* subfamilies except ζ which are sensitive to both water-soluble and airborne odorants (Niimura 2009), consistent with their aquatic and terrestrial lifecycles. The *L. oculatus* genome also contains all subfamilies of *OR* genes.

### Phylogenetic Generalized Least-Squares Regression Analysis

**Fig. 4.**
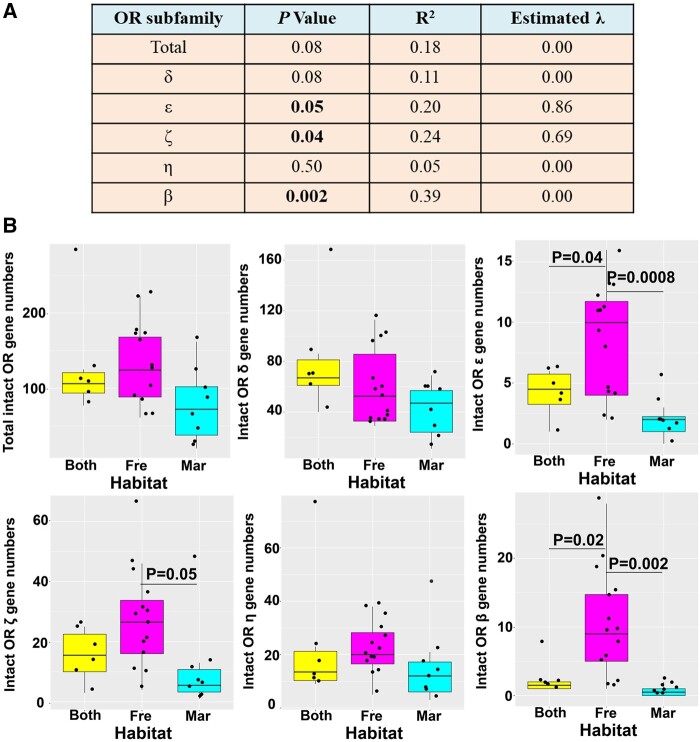
*OR* genes subfamilies were compared among fish with different habitats. (*A*) Phylogenetic generalized least squares (PGLS) regression analysis for number of intact *OR* genes in fish species (*n* = 28) versus ecological niches (habitats). (*B*) Box plot of intact *OR* gene numbers among groups of divergent ecological niches. One-way ANOVA analysis and Tukey HSD test were conducted to determine statistical difference among groups. Mar, marine; Fre, freshwater; Both, both in marine and freshwater.

To investigate whether *OR* repertoire size is related to the ecological factors, we performed a phylogenetic generalized least squares (PGLS) regression analyses. We used two ecological niche factors as predictor variables for this analysis: Including habitat (marine, freshwater [FW] and both marine and FW) and feeding habit (carnivorous, omnivorous, and herbivorous) (supplementary note 1, Supplementary Material online). Although the habitat was not a significant predictor of the total number of intact *OR* genes, we found a strong effect of habitat on the number of subfamily β (Pagel’s λ = 0, *P *=* *0.002), ε (Pagel’s λ = 0.86, *P *=* *0.05), and ζ (Pagel’s λ = 0.69, *P *=* *0.04) *OR* genes (fig. 4*A*). However, we did not find a significant association between the number of *OR* repertoires and feeding habits (supplementary fig. 3*A*, Supplementary Material online).

To further explore the difference of *OR* repertoires among specified niches groups, one-way ANOVA analysis was performed (fig. 4*B*). The results indicated that the numbers of subfamily β (*P *=* *0.002), ε (*P *=* *0.0008), and ζ (*P *=* *0.05) *OR* genes in FW species were significantly higher than that in marine fish species. Additionally, the number of subfamily β (*P *=* *0.02) and ε (*P *=* *0.04) genes in FW fish were also significantly higher than in fish living in both FW and marine water. For feeding habits, only the number of β *OR* genes in the herbivorous species was significantly higher than in carnivorous species (*P *=* *0.05) (supplementary fig. 3*B*, Supplementary Material online).

### Molecular Evolution of Subfamily β and ε

**Fig. 5.**
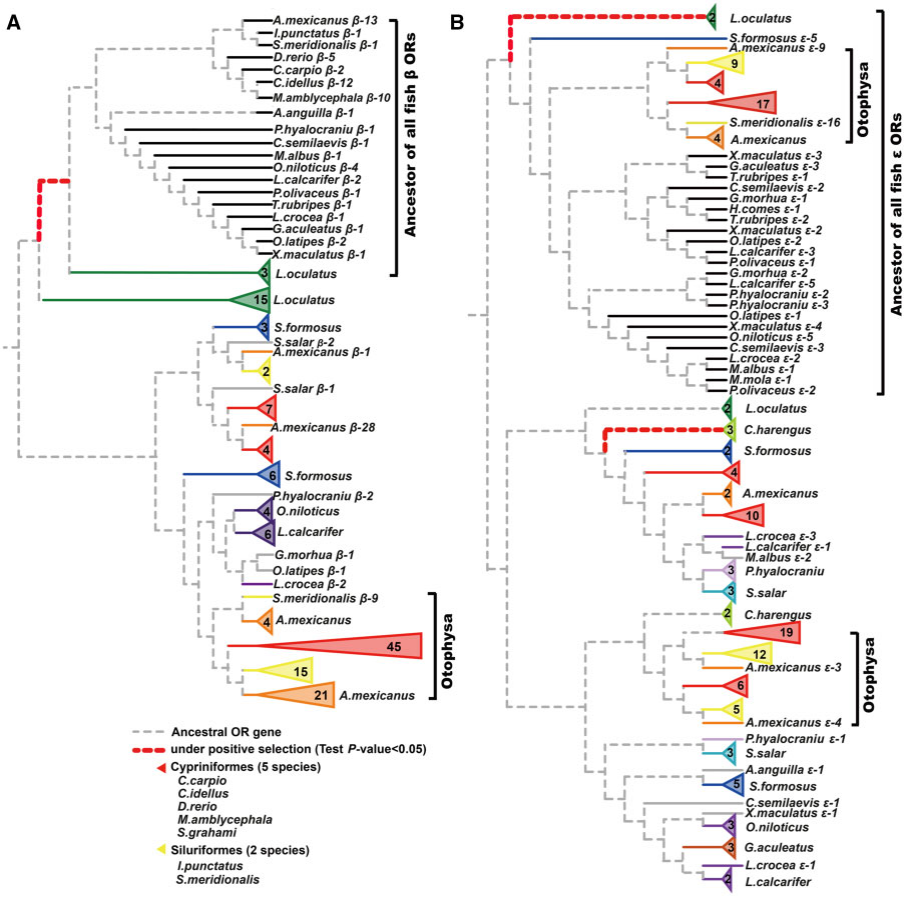
Molecular evolution of subfamily β (*A*) and ε (*B*) *OR* genes in 28 fish species. Fish from the same order are labeled with same color. The red bold dashed lines in the branches of the trees have *P *<* *0.05 (corrected for multiple testing) considered to have experienced diversifying positive selection.

Our phylogenetic analysis of 164 β and 161 ε *OR* genes in fish uncovered lineage-specific expansions in *M. amblycephala* and the other fish species of the Otophysa lineage (fig. 5; supplementary figs. 4 and 5, Supplementary Material online). The majority of fish species included in this study have one or two copies of β *OR* genes, whereas all Otophysa species show an expansion of the β subfamily (more than 10 on average). Specifically, multiple highly supported clades of β *OR* genes suggest recurrent and lineage-specific expansions for Otophysa β *ORs* (fig. 5*A*). Similarly, genes in subfamily ε also independently duplicated multiple times in the Otophysa lineage (fig. 5*B*). To test whether these duplicated copies are under selection, we used an adaptive branch-site random effects likelihood (aBSREL) model to explore the presence/absence of selection by measuring rates of nonsynonymous to synonymous (dN/dS = ω) substitutions in genes. We found that four and three branches show strong support being under significant positive selection (test *P*-value < 0.05) in β and ε ORs trees, respectively (fig. 5*A*;supplementary table 7, notes 2 and 3, Supplementary Material online). Of note, ORs of the subfamily β show evidence of positive selection in the ancestor of fish species included in this study, but not in branches that would represent lineage-specific expansions (fig. 5).

### Expression Patterns of OR Genes

**Fig. 6.**
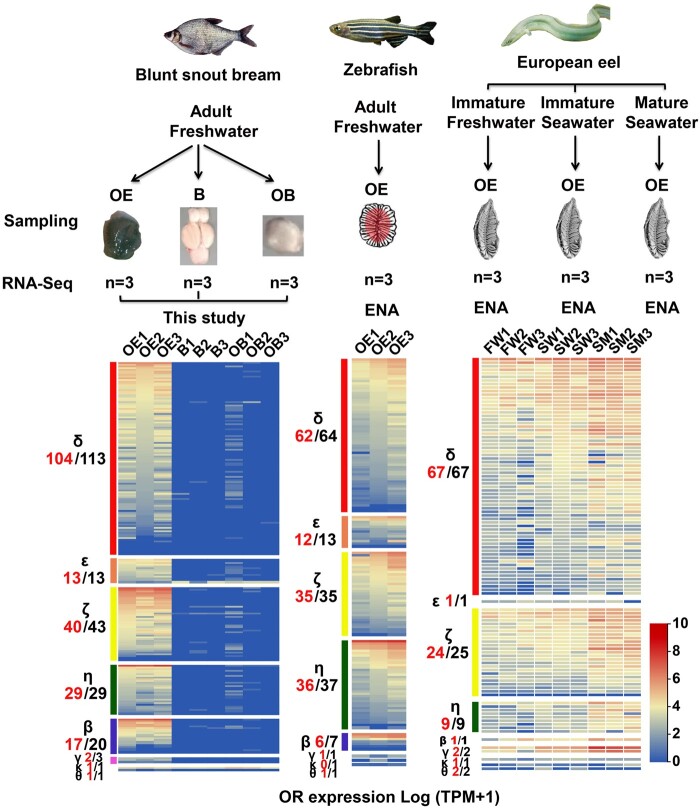
Gene expression profiles of *ORs* in *M. amblycephala*, *D. rerio* and *A. anguilla*. A total of nine sequencing libraries, including three olfactory epithelium (OE), three whole brain (B) and three olfactory bulb (OB) of *M. amblycephala* were sequenced. Three RNA-seq raw data of OE from adult *D. rerio and* nine OE from *A. anguilla* were also analyzed. RNA expression levels are represented on a log_2_(*X* + 1) scale of normalized TPM (0, not expressed; 10, highly expressed). *OR* genes in each subfamily are displayed in descending order of their expression values. Numbers labeled in red represent the number of expressed *OR* genes while numbers in black are the total number of intact genes in each subfamily.

To determine which of the *OR* genes are being used, we performed RNA-seq on the OE, whole brain (B), and olfactory bulb (OB) of adult *M. amblycephala* and analyzed previously reported OE transcriptome data from adult *D. rerio* and of different life stages of *A. anguilla* (fig. 6). Our results demonstrate that the majority of candidate *OR* genes are expressed in the OE (fig. 6). In *M. amblycephala*, *OR* genes were not expressed in brain and OB, whereas 207 of 223 intact *ORs* were expressed in OE. In *D. rerio*, 153 of 159 intact *ORs* were expressed in OE. In *A. anguilla*, all the *OR* genes were expressed in OE except one ζ subfamily. Importantly, the expression level of the majority *ORs* of *A. anguilla* were higher in sexually mature male (SM) than in sexually immature FW and seawater (SW) OE. These results suggest that most intact *ORs* were expressed and putatively functional in the OE of these species.

**Fig. 7.**
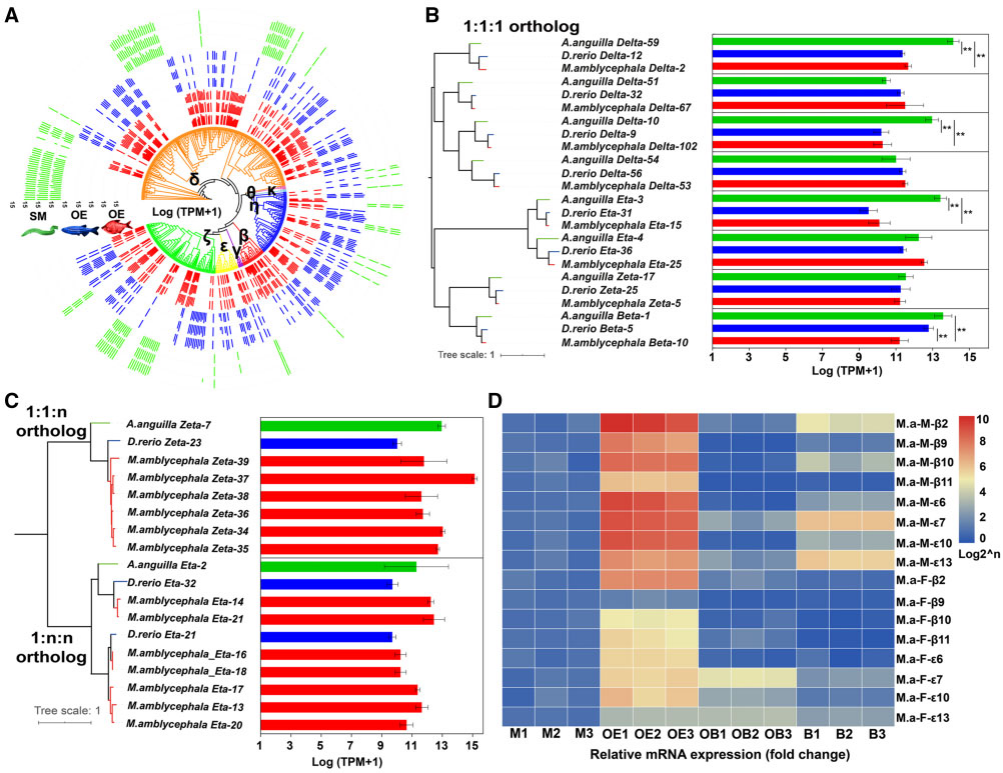
Comparison of *OR* genes expression patterns among different fish species. (*A*) Unrooted phylogenetic tree and normalized expression levels for all intact *OR* genes among *M. amblycephala*, *D. rerio* and *A. anguilla*. Bars indicate the relative expression level represented on a log_2_(X + 1) scale of normalized TPM. (*B*) and (*C*) Phylogenetic tree (left) and expression levels (right) of 1:1:1 and 1: n: n *OR* orthologs in *A. anguilla*, *D. rerio* and *M. amblycephala*. Bars showed the RNA-seq expression values (mean ± SD, *n* = 3) for the *OR* genes in fish OE. One-way ANOVA analysis and Tukey HSD test were conducted to determine statistical difference among species (**P <* *0.01). (*D*) The relative expression levels of representative β and ε *OR* genes detected by qRT-PCR. Expression in olfactory epithelium (OE), olfactory bulb (OB) and brain tissues (B) were normalized to the expression in muscle (M). M.a*-*M, male *M. amblycephala*; M.a*-*F, female *M. amblycephala.*

To evaluate whether there are differences in *OR* expression levels among the three species, we plotted phylogenetic trees generated from all intact OR sequences overlaid with the normalized values corresponding to their expression (fig. 7*A*). Half of eight identified 1:1:1 *OR* orthologs were expressed at significantly higher levels (*P *<* *0.01) in *A. anguilla* than in *M. amblycephala* OE (fig. 7*B*). Among the 1:1:n and 1:n:n orthologs, nearly all the duplicated *ORs* are highly expressed (fig. 7*C*). To further validate the transcriptome results and verify the functional significance of subfamily β and ε *OR* genes, we selected eight representative β and ε *ORs* in four tissues (muscle, OE, OB, and brain) of adult male and female *M. amblycephala* using real-time quantitative PCR (qRT-PCR) (fig. 7*D*). All the tested β and ε *OR* genes were highly expressed in OE of male and female *M. amblycephala* (fig. 7*D*), whereas these were not expressed in muscle tissue, OB and brain except β2 ε7, ε13 with slight expression in male *M. amblycephala* brain. The expression trend of these *ORs* revealed using qRT-PCR analysis was consistent with that detected in the OE transcriptome analysis.

## Discussion

Sensory systems play a crucial role in the aquatic lifestyle of fish in feeding, migration, spawning, and defense (Hara 1975). Gene expansion in *ORs* is thought to have played an important role in the transition from aquatic to terrestrial living in mammals (Niimura 2009; Hayden et al. 2010), but the role of *ORs* expansion among aquatic species has not been previously studied using comparative evolutionary analysis. Using a high-quality genome assembly of *M. amblycephala* and data from 27 taxonomically diverse fish species, we found that the numbers of intact *OR* genes have changed extensively during fish evolution. We find evidence for an expansion of *OR* genes in FW species, in particular as regards members of the β and ε subfamilies, which most likely reflects ecological adaptation.

Genomic surveys have revealed that *ORs* represent the largest gene family in mammals (Zhang and Firestein 2002) and some studies indicate that OR repertoires vary widely among vertebrates even between closely related taxa (Matsui et al. 2010; Niimura et al. 2014, 2018). The variation in the number of intact *OR* genes is >10-fold among the 28 teleosts examined in this study, ranging from 20 in *M. mola* to 279 in *L. calcarifer* (supplementary table 6, Supplementary Material online). This result is consistent with previous analyses of the total number of *OR* genes from the whole genome data of *T. rubripes* (44 *ORs*), *Tetraodon nigroviridis* (42 *ORs*) (Alioto and Ngai 2005), *D. rerio* (167 *ORs*), *Hippocampus comes* (26 *ORs*) (Lin et al. 2016), and *Larimichthys crocea* (112 *ORs*) (Ao et al. 2015). The MRCA between fish and tetrapods had at least nine subfamilies of *OR* genes (Niimura and Nei 2005) and at least six (δ, ε, ζ, η, β, and γ) out of the seven classified subfamilies of intact *OR* genes are retained in fish (fig. 3; supplementary table 6, Supplementary Material online). The pattern is opposite in mammals, where only two subfamilies (α and γ) are retained, but their copy numbers are greatly expanded (Niimura 2009).

Most fish possess a well-developed olfactory system and have a relatively large OB and OE. But why do the number of intact *OR* genes vary so much among fish species? We predicted that *OR* genes might vary according to different habitats and lifestyles. Gene duplications followed by subfunctionalization is one possible mechanism for genetic adaptation (Chang and Duda 2012). Comparative studies of *OR* gene families have demonstrated birth and death of genes as a model of OR evolution involving tandem duplications and chromosomal rearrangements (Niimura and Nei 2003; Nei and Rooney 2005; Hughes et al. 2018; McKenzie and Kronauer 2018). Analysis of three cyprinid fishes showed that fish ORs are found in several genomic clusters (supplementary fig. 2, Supplementary Material online). Within gene clusters, different subfamilies of *OR* genes are largely contiguous and located close to each other and with the same transcriptional orientation, suggesting tandem duplication as a mechanism of gene expansion, consistent with a previous fish study (Alioto and Ngai 2005).

Studies on reptiles, birds and mammals have suggested that expansion and contraction of *OR* genes are related to major changes in habitat and lifestyle (Niimura 2009; Hayden et al. 2010, 2014; Khan et al. 2015; Vandewege et al. 2016; Hughes et al. 2018). For example, previous study explored OR diversity in 2 reptiles and 48 birds, which indicated species and lineage-specific variation in subfamily of *OR* genes associated with aquatic and terrestrial adaptations (Khan et al. 2015). Similarly, a study on *OR* genes in sauropsida suggests that adaption related to life history evolution have shaped the unique OR repertoires in different members of this subfamily (Vandewege et al. 2016). Our taxonomically diverse sampling allowed us to assess the relationship between habitat and feeding behavior in relation to *OR* gene expansion in fish. Transitions to different aquatic environments may be facilitated by the olfactory sensory organs and *OR* genes if olfaction is critical to survival. For example, elimination of visual function resulted in increased olfactory capabilities in the blind cave fish, *A. mexicanus* (Blin et al. 2018). In the present study, we observed a substantial expansion of subfamily β *OR* genes in *A. mexicanus* genome, which may compensate to some extent their loss of visual information (supplementary table 6, Supplementary Material online).

Our phylogenetically controlled environmental association analysis (fig. 4) indicated that although the total number of intact *OR* genes was unaffected by environmental factors, there is a strong association between the number of subfamily β, ε, and ζ *OR* genes and FW habitat. Most teleosts possess one or two β *OR* genes, whereas fish in the Otophysa lineage possess more than 10 on average (fig. 5*A*;supplementary table 6, Supplementary Material online). Why might β *OR* genes have specifically expanded in FW Otophysa species? A possible explanation for this observation is that subfamily β *OR* genes are important for detecting both water soluble and airborne odorants (Niimura 2009). The large number of β and ε *OR* genes in the FW Otophysa lineage may allow them to detect and discriminate a wide range of odorant stimuli that are important for survival in FW. Previous research has suggested that expansion or contraction of gene families can be a random process driven by drift and/or by natural selection. Earlier studies highlighted that positive selection shaped the diversification and variation of the *OR* gene family in ants (Engsontia et al. 2015), bees (Brand and Ramírez 2017), and fish (Alioto and Ngai 2005; Hussain et al. 2009). However, in this study, no signatures of positive selection were found in the β and ε *ORs* showing copy number expansion (fig. 5).

Calculating total mRNA abundance of each OR permitted us to assess which receptors are functionally expressed in the OE transcriptomes of *M. amblycephala* and *D. rerio* (FW fish), as well as *A. anguilla* (both living in FW and SW). We present evidence that almost all the intact *OR* genes belonging to different subfamilies are expressed and putatively functional in OE of these species (fig. 6). Similar *OR* expression patterns have also been observed in the mouse (Ibarra-Soria et al. 2014) and human (Olender et al. 2016). Although the number of intact *ORs* (108) in *A. anguilla* is lower than in *D. rerio* (159 intact *ORs*) and in *M. amblycephala* (223 intact *ORs*), the expression levels of four out of eight 1:1:1 *OR* orthologs in SW stage *A. anguilla* were significantly higher than that in FW *M. amblycephala* (fig. 7*B*). Previous work describing differential expression of *OR* genes in OE of wild Atlantic salmon (Johnstone et al. 2011) and European eel (Churcher et al. 2015) suggests that the regulation of these genes is associated with different physiological states and responses to environmental cues.

In summary, we present the first phylogenetic comparative analysis of *OR* genes expansion and contraction in fish. We show that expansions of subfamily β and ε *OR* genes have occurred in the FW Otophysa lineage and that β and ε *OR* genes expansion is consistently associated with FW habitats in other species. Gene expression analyses indicated that nearly all the intact *OR* genes including expanded subfamily β and ε *OR* genes in FW fish are expressed in olfactory organs, strongly supporting their functional importance, which have potentially facilitated FW adaptation.

## Materials and Methods

### Genome Sequencing and Assembly

Genomic DNA was isolated from whole blood of a double haploid fish as described in our previous study (Liu et al. 2017). The genomic DNA was fragmented to 20 kb and sequenced with the Pacific Biosystems RSII platform. The genome assembly was performed using a combination of sequencing technologies: PacBio RS II reads, Illumina paired-end reads (PE), and Illumina mate-pair reads (MP). Briefly, high-quality Illumina PE reads were separately assembled into Illumina contigs using Platanus (v1.2.4) (Kajitani et al. 2014). Next, low-quality PacBio subreads with a read length shorter than 1,000 bp or a quality score lower than 0.8 were filtered out. The remaining PacBio subreads were error-corrected with MECAT (v 1.0) (Xiao et al. 2017). Then, the error-corrected PacBio reads and Illumina contigs were combined to perform a hybrid assembly using the DBG2OLC (Ye et al. 2016) pipeline. Illumina reads (65× coverage) were mapped to the contigs using BWA-aln. This alignment was then used to correct the assembly with Pilon 1.22 (Walker et al. 2014). A total of 69.15 Gb of Illumina MP data (approximately 61×), with an insert size varying between 2 and 20 kb, was used to scaffold the assembly with SSPACE (v3.0) (Boetzer et al. 2011). Then, the gaps were filled with GapFiller (v1.10) (Nadalin et al. 2012) and PBjelly (v1.2) (English et al. 2012). To further estimate the completeness of the assembly, Core Eukaryotic Genes Mapping Approach was performed using benchmarking universal single-copy orthologs (BUSCO) (SimAo et al. 2015).

### Chromosome-Level Assembly and Synteny Analyses between *M. amblycephala* and *D. rerio*

A RAD genetic linkage map of *M. amblycephala* (Liu et al. 2017) composed of 14,648 SNP markers was used to organize and orientate the scaffolds into chromosome-sized sequences. The RAD tags corresponding to these 14,648 SNP markers were mapped onto the genome using BWA-aln. Then, we placed and oriented the scaffolds and contigs relative to each other with the physical and genetic positions of the mapped markers. To identify syntenic blocks, the protein sequences from *M. amblycephala* and *D. rerio* were searched against each other using BLASTp (E < 1e−5). The results were subjected to MCScan (–a, –e : 1e–5, –u : 1, –s : 5) to determine syntenic blocks.

### Genome Annotation

We used a combination of homology-based, de novo and RNA-seq annotation methods to predict genes in the assembled genome. For the sequence similarity-based prediction, protein sequences from *D. rerio*, *G. aculeatus*, *L. crocea*, *L. calcarifer*, *L. oculatus*, *O. niloticus*, *O. latipes*, and *S. grahami* were mapped to *M. amblycephala* genome using genBlastA v1.0.1 (She et al. 2009) with an E-value threshold of 1e−5. Subsequently, homologous genome sequences were aligned against the matched proteins to define gene models using GeneWise (v2.2.0) (Birney et al. 2004). For the de novo gene predictions, AUGUSTUS (v2.5.5) (Stanke et al. 2006) was used to identify candidate protein-encoding genes in the masked genome with self-trained model parameters. Then unigene sequences from nine transcriptoms sequenced this study were used to search transcripts region using BLAT v. 36 and PASA (Haas et al. 2003). Finally, the homology-based, de novo-derived and transcript gene sets were merged to generate a high confidence gene set using Maker 2.31.10 (Holt and Yandell 2011). To assign functions to the gene models, we performed BLASTP, with an E-value threshold of ≤10^−5^, using the NR, KOG, SwissProt, and TrEMBL databases (Uniprot release 2017-09). The gene motifs and domains were determined using InterProScan (version 5.16) (Zdobnov and Apweiler 2001) against public protein databases. Functional annotation was carried out using Blast2GO (Conesa et al. 2005).

### Phylogenetic Tree Construction and Divergence Time Estimation

We first extracted 22,708 orthogroups conserved among 28 species (*C. harengus*, *C. idellus*, *C. semilaevis*, *C. carpio*, *D. rerio*, *G. morhua*, *G. aculeatus*, *H. comes*, *I. punctatus*, *L. crocea*, *L. calcarifer*, *L. chalumnae*, *L. oculatus*, *M. amblycephala*, *M. mola*, *M. albus*, *O. niloticus*, *O. latipes, P. olivaceus*, *P. hyalocraniu*, *S. salar*, *S. meridionalis*, *S. grahami*, *T. rubripes*, *X. maculatus*, *A. anguilla, A. mexicanus*, and *S. formosus*) using OrthoFinder v2.3.11 (Emms and Kelly 2019). Of these, 41 single copy gene families were detected by the following steps. Firstly, we did pairwise alignment among protein sequences through Diamond v0.9.24.125 (Buchfink et al. 2014). Secondly, we use MCL software to cluster the pairwise comparison of protein sequences, do multiply alignment with Mafft (Katoh and Standley 2013) and construct phylogeny tree of each orthogroups through FastTree2 (Price et al. 2010). At last, we set the root by Species Tree Root Inference from Duplication (STRIDE) (Emms and Kelly 2017). Divergence times were calculated by the PAML mcmctree program (Reis and Yang 2011). The parameters were set as the following: the approximate likelihood calculation method, the correlated molecular clock, REV substitution model. And three fossil calibration time used to calculate divergence times of the other branch were: *L. chalumnae* versus *D. rerio* 416 − 422 Ma; *D. rerio* versus *G. aculeatus*: 149.85 − 165.2 Ma, *O. latipes* vs *G. aculeatus*: 96.9 − 150.9 Ma.

### Identification of OR Genes from Fish Genome Sequences

To identify *OR* genes from 28 complete fishes genome (supplementary table 5, Supplementary Material online), we followed a bioinformatics pipeline similar to previously described methods (Niimura and Nei 2007; Niimura 2009) with some modifications. Briefly, we used a first-round of TBLASTN searches (Altschul et al. 1997) with a cutoff E-value of 1e−5 against each fish genome sequence using a set of known functional OR genes sequences from *D. rerio*, *O. latipes*, *O. niloticus*, *L. chalumnae*, *L. oculatus*, *L. vexillifer*, and *T. rubripes* as queries. We then predicted the structure of the *OR* genes using the blast-hit sequence with GeneWise (Birney et al. 2004), extending in both 3’ and 5’ directions along the genome sequences. Hits shorter than 200 bp were discarded. All best-hits from the genome sequence were extracted. We classified *OR* genes with interrupting stop codons or frame-shifts were as pseudogenes. We classified a truncated gene as those with a partially intact sequence encoding a part of an *OR* and were validated by alignment to functional genes using the program MUSCLE (Edgar 2004). The longest coding sequences from the start (ATG) codon to a stop codon with an uninterrupted open reading frame and seven transmembrane domains were considered as intact *OR* genes. To classify *OR* genes into different subfamilies, we used BLASTP to intact *ORs* into putative subfamilies based on the classification of zebrafish and pufferfish *OR* genes (Niimura and Nei 2005). A phylogenetic tree was constructed using the ML method in MEGA 5.10 (Tamura et al. 2011) with 1,000 replicates to verify and correct the putative BLASTP-based assignments. Finally, we aligned all identified intact *OR* genes found in this study using MUSCLE. The alignment was manually corrected and used to construct a phylogenetic tree by FastTree2 (Price et al. 2010). The phylogenetic tree was displayed and labeled using Interactive Tree Of Life (iTOL) v4 (Letunic and Bork 2019). All the identified intact and truncated OR nucleic and translated protein sequences in this study are shown in supplementary notes 4 and 5, Supplementary Material online.

### Collinear Analysis of OR Genes Among *M. amblycephala*, *D. rerio*, and *C. idellus*

Syntenic blocks between *M. amblycephala* and *D. rerio*, and between *M. amblycephala* and *C. idellus* were firstly identified using BLASTp and MCScan following methods described above. Then, the *OR* genes from these three fish were positioned back on their genome to determine which genes belong to which syntenic region. Finally, variant sites among *OR* genes located in syntenic region were identified by multiple sequence alignment using MUSCLE.

### Estimation of OR Genes Gain and Loss Events

We used CAFE′ (De Bie et al. 2006) to reconstruct the OR repertoires and calculate copy numbers for ancestral *OR* genes in each lineage using all the intact *ORs* identified from fish genomes. Firstly, a data file containing the sizes of all the *OR* gene subfamilies were prepared. Divergence times for each node in the CAFE′ analyses were estimated from the phylogenetic tree (fig. 2*A*). The CAFE′ method employs a random birth and death model to estimate gene gains and losses in each lineage. The global parameter λ described both the gene birth (λ) and death (μ = −λ) rate across all branches in the tree for all gene subfamilies was estimated using ML. A conditional *P*-value was calculated for each gene family, and families with conditional *P*-values of <0.05 were considered to have a notable gain or loss.

### Molecular Evolution and Selection Analyses of Subfamily β and ε OR Genes

All intact subfamily β and ε *OR* genes from 28 fish genomes (β, *n* = 164 and ε, *n* = 161) were aligned to prepare the alignments. The *OR* genes trees were constructed using the ML method in MEGA 5.10. We used the aBSREL approach (Smith et al. 2015) in order to test for evidence of episodic positive selection implemented in Datamonkey (Weaver et al. 2018). In aBSREL, a likelihood ratio test was performed to compare the null model (ω = 1) against the alternative, where the branch was undergoing some form of selection (ω ≠ 1). We used a threshold of *P *<* *0.05 (after correction for multiple testing) to infer positive selection at the marked branches.

### PGLS Regression Analysis

We use phylogenetic generalized least-squares regressions (PGLSs) to establish the relationships between the numbers of *OR* genes and ecological factors (feeding habit and habitat) of each fish species while controlling for phylogenetic effects. Feeding habit was categorized as carnivorous, omnivorous or herbivorous and habitat was categorized as marine, FW , or both marine and FW (supplementary note 1, Supplementary Material online). The PGLS analyses were performed using the R packages “caper” (Orme 2013). The input phylogenetic tree was from our present study (fig. 2*A*). Here, we used Pagel’s λ (Pagel 1999) with the value ranging from 0 (phylogenetic independence) to 1 (phylogenetic dependence) to estimate the degree of phylogenetic signal of each trait.

### RNA-Seq Analysis

The experimental procedures were approved by the Animal Care and Use Committee of Huazhong Agricultural University. The OE, OB, and whole brain (B) were collected from the adult male *M. amblycephala* and immediately frozen in liquid nitrogen. All samples were prepared in triplicate. Total RNA was isolated from each sample with RNAiso Plus (TaKaRa, Dalian, China). A total of nine RNA-seq libraries were constructed and sequenced in BGI by BGISEQ-500 platform (Shenzhen, China). For comparison, the raw data from OE of adult *D. rerio* and different life stages, FW, SW, and SM of *A. anguilla* were downloaded for further analyses. The detailed sampling information is shown in supplementary table 8, Supplementary Material online.

All raw data were assessed using fastp v0.20.0 (Chen et al. 2018). After removing the adapters and poly N or low-quality sequences (*Q* < 15), the remainder were termed as clean reads. High quality clean reads from each sample were separately aligned to *M. amblycephala* (this study), *D. rerio* (GRCz11.99, Ensembl) and *A. anguilla* (GCA_000695075.1, NCBI) genome using Hitsat2 v2.0.4 (Pertea et al. 2016). Estimated mapped read counts and transcript lengths were used to calculate transcripts per million (TPM) using RSEM (v1.2.25) with the default settings (Li and Dewey 2011). To reduce the variation among the three fish species, the DESeq2 package (Love et al. 2014) was also used to estimate the size factors and dispersion and to generate a normalized counts matrix using the 4,404 single-copy orthologs. TPM values were subsequently transformed to log_2_ (TPM + 1).

### qRT-PCR Analysis

A total of 8 representative β and ε *OR* genes in four tissues (muscle, OE, OB, and B) of adult male and female *M. amblycephala* were detected by using qRT-PCR. The OE, OB, B, and muscle tissues were collected from the adult male (*n* = 9) and female *M. amblycephala* (*n* = 9) and immediately frozen in liquid nitrogen. Tissues from three individuals were pooled to extract RNA and three independent biological replicates for male and female were separately prepared. cDNA was synthesized from 1 μg of total RNA using a reverse transcriptase kit from TaKaRa Biochemicals (TaKaRa, Dalian, China). Primers were designed using Primer 5.0 software (supplementary table 9, Supplementary Material online). β-actin served as an internal normalization control for qRT-PCR analysis. PCR reactions contain 1 μl cDNA, 1 μl forward and reverse primers, 10 μl SYBR Green PCR Master Mix (TaKaRa, Dalian, China). A qRT-PCR was performed using a Roter-gene Q (Qiagen, Hilden, Germany) with one cycle of predenaturation at 95 °C for 45 s, followed by 40 cycles of amplification at 95 °C for 15 s, 60 °C for 15 s, and 72 °C for 30 s.

## Supplementary Material

Supplementary data are available at *Molecular Biology and Evolution* online.

## Supplementary Material

msab152_Supplementary_Data

## References

[msab152-B1] Alioto TS, Ngai J. 2005. The odorant receptor repertoire of teleost fish. BMC Genomics 6:173. 16332259 10.1186/1471-2164-6-173 PMC1325023

[msab152-B2] Altschul SF, Madden TL, Schaffer AA, Zhang J, Zhang Z, Miller W, Lipman DJ. 1997. Gapped BLAST and PSI-BLAST: a new generation of protein database search programs. Nucleic Acids Res. 25:3389–3402. 9254694 10.1093/nar/25.17.3389 PMC146917

[msab152-B3] Ao J, Mu Y, Xiang LX, Fan D, Feng M, Zhang S, Shi Q, Zhu L-Y, Li T, Ding Y, et al 2015. Genome sequencing of the perciform fish *Larimichthys crocea* provides insights into molecular and genetic mechanisms of stress adaptation. PLoS Genet. 11:e1005118. 25835551 10.1371/journal.pgen.1005118 PMC4383535

[msab152-B4] Bazáes A, Olivares J, Schmachtenberg O. 2013. Properties, projections, and tuning of teleost olfactory receptor neurons. J Chem Ecol. 39:451–464. 23468224 10.1007/s10886-013-0268-1

[msab152-B5] Birney E, Clamp M, Durbin R. 2004. GeneWise and Genomewise. Genome Res. 14:988–995. 15123596 10.1101/gr.1865504 PMC479130

[msab152-B6] Blin M, Tine E, Meister L, Elipot Y, Bibliowicz J, Espinasa L, Rétaux S. 2018. Developmental evolution and developmental plasticity of the olfactory epithelium and olfactory skills in Mexican cavefish. Dev Biol. 441:242–251. 29709597 10.1016/j.ydbio.2018.04.019

[msab152-B7] Boetzer M, Henkel CV, Jansen HJ, Butler D, Pirovano W. 2011. Scaffolding pre-assembled contigs using SSPACE. Bioinformatics 27:578–579. 21149342 10.1093/bioinformatics/btq683

[msab152-B8] Brand P, Ramírez SR. 2017. The evolutionary dynamics of the odorant receptor gene family in corbiculate bees. Genome Biol Evol. 9:2023–2036. 28854688 10.1093/gbe/evx149 PMC5597890

[msab152-B9] Buchfink B, Xie C, Huson DH. 2014. Fast and sensitive protein alignment using DIAMOND. Nat Methods. 12:59–60. 25402007 10.1038/nmeth.3176

[msab152-B10] Chang D, Duda TF Jr. 2012. Extensive and continuous duplication facilitates rapid evolution and diversification of gene families. Mol Biol Evol. 29:2019–2029. 22337864 10.1093/molbev/mss068

[msab152-B11] Chen S, Zhou Y, Chen Y, Gu J. 2018. fastp: an ultra-fast all-in-one FASTQ preprocessor. Bioinformatics 34:i884–i890. 30423086 10.1093/bioinformatics/bty560 PMC6129281

[msab152-B12] Churcher AM, Hubbard PC, Marques JP, Canário AVM, Huertas M. 2015. Deep sequencing of the olfactory epithelium reveals specific chemosensory receptors are expressed at sexual maturity in the European eel *Anguilla*. Mol Ecol. 24:822–834. 25580852 10.1111/mec.13065

[msab152-B13] Conesa A, Götz S, García-Gómez JM, Terol J, Talón M, Robles M. 2005. Blast2GO: a universal tool for annotation, visualization and analysis in functional genomics research. Bioinformatics 21:3674–3676. 16081474 10.1093/bioinformatics/bti610

[msab152-B14] Cong X, Zheng Q, Ren W, Chéron JB, Fiorucci S, Wen T, Zhang C, Yu H, Golebiowski J, Yu Y. 2019. Zebrafish olfactory receptors ORAs differentially detect bile acids and bile salts. J Biol Chem. 294:6762–6771. 30833327 10.1074/jbc.RA118.006483 PMC6497948

[msab152-B15] De Bie T, Cristianini N, Demuth JP, Hahn MW. 2006. CAFE: a computational tool for the study of gene family evolution. Bioinformatics. 22:1269–1271. 16543274 10.1093/bioinformatics/btl097

[msab152-B16] Edgar RC. 2004. MUSCLE: multiple sequence alignment with high accuracy and high throughput. Nucleic Acids Res. 32:1792–1797. 15034147 10.1093/nar/gkh340 PMC390337

[msab152-B17] Emms DM, Kelly S. 2017. STRIDE: species tree root inference from gene duplication events. Mol Biol Evol. 34:3267–3278. 29029342 10.1093/molbev/msx259 PMC5850722

[msab152-B18] Emms DM, Kelly S. 2019. OrthoFinder: phylogenetic orthology inference for comparative genomics. Genome Biol. 20(1):14. 31727128 10.1186/s13059-019-1832-y PMC6857279

[msab152-B19] English AC, Richards S, Han Y, Wang M, Vee V, Qu J, Qin X, Muzny DM, Reid JG, Worley KC, et al 2012. Mind the gap: upgrading genomes with Pacific biosciences RS long-read sequencing technology. PLoS One 7:e47768. 23185243 10.1371/journal.pone.0047768 PMC3504050

[msab152-B20] Engsontia P, Sangket U, Robertson HM, Satasook C. 2015. Diversification of the ant odorant receptor gene family and positive selection on candidate cuticular hydrocarbon receptors. BMC Res Notes. 8:380. 26306879 10.1186/s13104-015-1371-x PMC4549895

[msab152-B21] Haas BJ, Delcher AL, Mount SM, Wortman JR, Smith RK, Hannick LI, Maiti R, Ronning CM, Rusch DB, Town CD, et al 2003. Improving the *Arabidopsis* genome annotation using maximal transcript alignment assemblies. Nucleic Acids Res. 31:5654–5666. 14500829 10.1093/nar/gkg770 PMC206470

[msab152-B22] Hamdani EH, Døving KB. 2007. The functional organization of the fish olfactory system. Prog Neurobiol. 82:80–86. 17433527 10.1016/j.pneurobio.2007.02.007

[msab152-B23] Hara TJ, Zhang C. 1996. Spatial projections to the olfactory bulb of functionally distinct and randomly distributed primary neurons in salmonid fishes. Neurosci Res. 26(1):65–74. 8895893 10.1016/0168-0102(96)01078-4

[msab152-B24] Hara TJ. 1975. Olfaction in fish. Prog Neurobiol. 5:271–335. 830087 10.1016/0301-0082(75)90014-3

[msab152-B25] Hayden S, Bekaert M, Crider TA, Mariani S, Murphy WJ, Teeling EC. 2010. Ecological adaptation determines functional mammalian olfactory subgenomes. Genome Res. 20:1–9. 19952139 10.1101/gr.099416.109 PMC2798820

[msab152-B26] Hayden S, Bekaert M, Goodbla A, Murphy WJ, Dávalos LM, Teeling EC. 2014. A cluster of olfactory receptor genes linked to frugivory in bats. Mol Biol Evol. 31:917–927. 24441035 10.1093/molbev/msu043

[msab152-B27] Holt C, Yandell M. 2011. MAKER2: an annotation pipeline and genome-database management tool for second-generation genome projects. BMC Bioinformatics 12:491–491. 22192575 10.1186/1471-2105-12-491 PMC3280279

[msab152-B28] Hughes GM, Boston ES, Finarelli JA, Murphy WJ, Higgins DG, Teeling EC. 2018. The birth and death of olfactory receptor gene families in Mammalian niche adaptation. Mol Biol Evol. 35:1390–1406. 29562344 10.1093/molbev/msy028 PMC5967467

[msab152-B29] Hussain A, Saraiva LR, Korsching SI. 2009. Positive Darwinian selection and the birth of an olfactory receptor clade in teleosts. Proc Natl Acad Sci U S A. 106:4313–4318. 19237578 10.1073/pnas.0803229106 PMC2657432

[msab152-B30] Ibarra-Soria X, Levitin MO, Saraiva LR, Logan DW. 2014. The olfactory transcriptomes of mice. PLoS Genet. 10:e1004593. 25187969 10.1371/journal.pgen.1004593 PMC4154679

[msab152-B31] Johnstone KA, Lubieniecki KP, Koop BF, Davidson WS. 2011. Expression of olfactory receptors in different life stages and life histories of wild Atlantic salmon (*Salmo salar*). Mol Ecol. 20(19):4059–4069. 21883590 10.1111/j.1365-294X.2011.05251.x

[msab152-B32] Kajitani R, Toshimoto K, Noguchi H, Toyoda A, Ogura Y, Okuno M, Yabana M, Harada M, Nagayasu E, Maruyama H, et al 2014. Efficient de novo assembly of highly heterozygous genomes from whole-genome shotgun short reads. Genome Res. 24:1384–1395. 24755901 10.1101/gr.170720.113 PMC4120091

[msab152-B33] Katoh K, Standley DM. 2013. MAFFT multiple sequence alignment software version 7: improvements in performance and usability. Mol Biol Evol. 30:772–780. 23329690 10.1093/molbev/mst010 PMC3603318

[msab152-B34] Khan I, Yang Z, Maldonado E, Li C, Zhang G, Gilbert MTP, Jarvis ED, O’brien SJ, Johnson WE, Antunes A. 2015. Olfactory receptor subgenomes linked with broad ecological adaptations in sauropsida. Mol Biol Evol. 32:2832–2843. 26219582 10.1093/molbev/msv155

[msab152-B35] Laberge F, Hara TJ. 2001. Neurobiology of fish olfaction: a review. Brain Res Rev. 36:46–59. 11516772 10.1016/s0165-0173(01)00064-9

[msab152-B36] Letunic I, Bork P. 2019. Interactive Tree Of Life (iTOL) v4: recent updates and new developments. Nucleic Acids Res. 47:W256–W259. 30931475 10.1093/nar/gkz239 PMC6602468

[msab152-B37] Li B, Dewey CN. 2011. RSEM: accurate transcript quantification from RNA-Seq data with or without a reference genome. BMC Bioinformatics 12:323. 21816040 10.1186/1471-2105-12-323 PMC3163565

[msab152-B38] Lin Q, Fan S, Zhang Y, Xu M, Zhang H, Yang Y, Lee AP, Woltering JM, Ravi V, Gunter HM, et al 2016. The seahorse genome and the evolution of its specialized morphology. Nature 540:395–399. 27974754 10.1038/nature20595 PMC8127814

[msab152-B39] Liu H, Chen C, Gao Z, Min J, Gu Y, Jian J, Jiang X, Cai H, Ebersberger I, Xu M, et al 2017. The draft genome of blunt snout bream (*Megalobrama amblycephala*) reveals the development of intermuscular bone and adaptation to herbivorous diet. Gigascience 6:gix039. 10.1093/gigascience/gix039 PMC5570040 28535200

[msab152-B40] Love MI, Huber W, Anders S. 2014. Moderated estimation of fold change and dispersion for RNA-seq data with DESeq2. Genome Biol. 15:550. 25516281 10.1186/s13059-014-0550-8 PMC4302049

[msab152-B41] Matsui A, Go Y, Niimura Y. 2010. Degeneration of olfactory receptor gene repertories in primates: no direct link to full trichromatic vision. Mol Biol Evol. 27:1192–1200. 20061342 10.1093/molbev/msq003

[msab152-B42] McKenzie SK, Kronauer DJ. 2018. The genomic architecture and molecular evolution of ant odorant receptors. Genome Res. 28:1757–1765. 30249741 10.1101/gr.237123.118 PMC6211649

[msab152-B43] Mombaerts P. 1999. Seven transmembrane proteins as odorant and chemosensory receptors. Science 286:707–711. 10531047 10.1126/science.286.5440.707

[msab152-B44] Nadalin F, Vezzi F, Policriti A. 2012. GapFiller: a de novo assembly approach to fill the gap within paired reads. BMC Bioinformatics 13:S14. 10.1186/1471-2105-13-S14-S8 PMC3439727 23095524

[msab152-B45] Nei M, Rooney AP. 2005. Concerted and birth-and-death evolution of multigene families. Annu Rev Genet. 39:121–152. 16285855 10.1146/annurev.genet.39.073003.112240 PMC1464479

[msab152-B46] Niimura Y, Matsui A, Touhara K. 2014. Extreme expansion of the olfactory receptor gene repertoire in African elephants and evolutionary dynamics of orthologous gene groups in 13 placental mammals. Genome Res. 24:1485–1496. 25053675 10.1101/gr.169532.113 PMC4158756

[msab152-B47] Niimura Y, Matsui A, Touhara K. 2018. Acceleration of olfactory receptor gene loss in primate evolution: possible link to anatomical change in sensory systems and dietary transition. Mol Biol Evol. 35:1437–1450. 29659972 10.1093/molbev/msy042

[msab152-B48] Niimura Y, Nei M. 2003. Evolution of olfactory receptor genes in the human genome. Proc Natl Acad Sci U S A. 100:12235–12240. 14507991 10.1073/pnas.1635157100 PMC218742

[msab152-B49] Niimura Y, Nei M. 2005. Evolutionary dynamics of olfactory receptor genes in fishes and tetrapods. Proc Natl Acad Sci U S A. 102:6039–6044. 15824306 10.1073/pnas.0501922102 PMC1087945

[msab152-B50] Niimura Y, Nei M. 2007. Extensive gains and losses of olfactory receptor genes in mammalian evolution. PLoS One 2(8):e708. 17684554 10.1371/journal.pone.0000708 PMC1933591

[msab152-B51] Niimura Y. 2009. On the origin and evolution of vertebrate olfactory receptor genes: comparative genome analysis among 23 chordate species. Genome Biol Evol. 1:34–44. 20333175 10.1093/gbe/evp003 PMC2817399

[msab152-B52] Olender T, Keydar I, Pinto JM, Tatarskyy P, Alkelai A, Chien MS, Fishilevich S, Restrepo D, Matsunami H, Gilad Y, et al 2016. The human olfactory transcriptome. BMC Genomics 17(1):18. 27515280 10.1186/s12864-016-2960-3 PMC4982115

[msab152-B53] Orme D, Freckleton R, Thomas G, Petzoldt T. 2013. The caper package: comparative analysis of phylogenetics and evolution in R. R Package Version 5:1–36.

[msab152-B54] Pagel M. 1999. Inferring the historical patterns of biological evolution. Nature 401(6756):877–884. 10553904 10.1038/44766

[msab152-B55] Pertea M, Kim D, Pertea GM, Leek JT, Salzberg SL. 2016. Transcript-level expression analysis of RNA-seq experiments with HISAT, StringTie and Ballgown. Nat Protoc. 11:1650–1667. 27560171 10.1038/nprot.2016.095 PMC5032908

[msab152-B56] Price MN, Dehal PS, Arkin AP. 2010. FastTree 2 - approximately maximum-likelihood trees for large alignments. PLoS One 5:e9490. 20224823 10.1371/journal.pone.0009490 PMC2835736

[msab152-B57] Reis MD, Yang Z. 2011. Approximate likelihood calculation on a phylogeny for Bayesian estimation of divergence times. Mol Biol Evol. 28:2161–2172. 21310946 10.1093/molbev/msr045

[msab152-B58] She R, Chu JSC, Wang K, Pei J, Chen N. 2009. genBlastA: enabling BLAST to identify homologous gene sequences. Genome Res. 19:143–149. 18838612 10.1101/gr.082081.108 PMC2612959

[msab152-B59] Simao FA, Waterhouse RM, Ioannidis P, Kriventseva EV, Zdobnov EM. 2015. BUSCO online supplementary information: assessing genome assembly and annotation completeness with single-copy orthologs. Bioinformatics 31:3210–3212. 26059717 10.1093/bioinformatics/btv351

[msab152-B60] Smith MD, Wertheim JO, Weaver S, Murrell B, Scheffler K, Kosakovsky Pond SL. 2015. Less is more: an adaptive branch-site random effects model for efficient detection of episodic diversifying selection. Mol Biol Evol. 32:1342–1353. 25697341 10.1093/molbev/msv022 PMC4408413

[msab152-B61] Stanke M, Keller O, Gunduz I, Hayes A, Waack S, Morgenstern B. 2006. AUGUSTUS: a b initio prediction of alternative transcripts. Nucleic Acids Res. 34:435–439. 10.1093/nar/gkl200 PMC1538822 16845043

[msab152-B62] Su CY, Menuz K, Carlson JR. 2009. Olfactory perception: receptors, cells, and circuits. Cell 139:45–59. 19804753 10.1016/j.cell.2009.09.015 PMC2765334

[msab152-B63] Tamura K, Peterson D, Peterson N, Stecher G, Nei M, Kumar S. 2011. MEGA5: molecular evolutionary genetics analysis using maximum likelihood, evolutionary distance, and maximum parsimony methods. Mol Biol Evol. 28:2731–2739. 21546353 10.1093/molbev/msr121 PMC3203626

[msab152-B64] van der Linden C, Jakob S, Gupta P, Dulac C, Santoro SW. 2018. Sex separation induces differences in the olfactory sensory receptor repertoires of male and female mice. Nat Commun. 9:5081. 30514924 10.1038/s41467-018-07120-1 PMC6279840

[msab152-B65] Vandewege MW, Mangum SF, Gabaldón T, Castoe TA, Ray DA, Hoffmann FG. 2016. Contrasting patterns of evolutionary diversification in the olfactory repertoires of reptile and bird genomes. Genome Biol Evol. 8:470–480. 26865070 10.1093/gbe/evw013 PMC4825420

[msab152-B66] Vassar R, Ngai J, Axel R. 1993. Spatial segregation of odorant receptor expression in the mammalian olfactory epithelium. Cell 74:309–318. 8343958 10.1016/0092-8674(93)90422-m

[msab152-B67] Walker BJ, Abeel T, Shea T, Priest M, Abouelliel A, Sakthikumar S, Cuomo CA, Zeng Q, Wortman J, Young SK, et al 2014. Pilon: an integrated tool for comprehensive microbial variant detection and genome assembly improvement. PLoS One 9:e112963. 25409509 10.1371/journal.pone.0112963 PMC4237348

[msab152-B68] Weaver S, Shank SD, Spielman SJ, Li M, Muse SV, Kosakovsky Pond SL. 2018. Datamonkey 2.0: a modern web application for characterizing selective and other evolutionary processes. Mol Biol Evol. 35:773–777. 29301006 10.1093/molbev/msx335 PMC5850112

[msab152-B69] Xiao CL, Chen Y, Xie SQ, Chen KN, Wang Y, Han Y, Luo F, Xie Z. 2017. MECAT: fast mapping, error correction, and de novo assembly for single-molecule sequencing reads. Nat Methods. 14:1072–1074. 28945707 10.1038/nmeth.4432

[msab152-B70] Ye C, Hill CM, Wu S, Ruan J, Ma ZS. 2016. DBG2OLC: efficient assembly of large genomes using long erroneous reads of the third generation sequencing technologies. Sci Rep. 6:31900. 27573208 10.1038/srep31900 PMC5004134

[msab152-B71] Zdobnov EM, Apweiler R. 2001. InterProScan–an integration platform for the signature-recognition methods in InterPro. Bioinformatics 17:847–848. 11590104 10.1093/bioinformatics/17.9.847

[msab152-B72] Zhang X, Firestein S. 2002. The olfactory receptor gene superfamily of the mouse. Nat Neurosci. 5:124–133. 11802173 10.1038/nn800

