# Supplementary material for: A Chromosome-Level Assembly of Blunt Snout Bream (Megalobrama amblycephala) Genome Reveals an Expansion of Olfactory Receptor Genes in Freshwater Fish
Source: Mol Biol Evol. 2021 May 18;38(10):4238–51. doi: 10.1093/molbev/msab152 (PMC8476165; doi:10.1093/molbev/msab152)
Supplement: msab152_Supplementary_Data [file msab152_supplementary_data.zip › 2 Supplementary Tables and Figures.docx]

**Supplementary Material**

**List of Supplementary Tables**

[Table 1 Pacific BioSciences raw read statistics. 2](#_Toc67649475)

[Table 2 Statistics of genome assembly. 3](#_Toc67649476)

[Table 3 Genome assembly completeness using BUSCO. 4](#_Toc67649477)

[Table 4 Scaffolding statistics based on linkage map data for each chromosome. 5](#_Toc67649478)

[Table 5 28 fish genome information. 6](#_Toc67649479)

[Table 6 Comparison of intact OR genes in 28 fish and other vertebrates. 7](#_Toc67649480)

[Table 7 Results from aBSREL analysis applied to Fig.5 9](#_Toc67649481)

[Table 8 Sampling information for RNA-Seq and qRT-PCR in this study. OB, olfactory bulb; B, brain, OE, olfactory epithelium. 10](#_Toc67649482)

[Table 9 Primers of representative β and ε OR genes in *M. amblycephala* for qRT-PCR. 11](#_Toc67649483)

**List of Supplementary Figures**

[Fig. 1. ML tree of the intact OR gene families identified across 28 fish genomes (n = 3,253). 12](#_Toc67649484)

[Fig. 2. Physical maps of intact OR genes in several genomic clusters among *D. rerio*, *C. idellus* and *M. amblycephala*. 15](#_Toc67649485)

[Fig. 3. OR genes subfamilies were compared among fish with different feeding habits. 16](#_Toc67649486)

[Fig. 4. Diversification of intact subfamily β (A) and ε (B) OR genes in the Otophysa lineage. Paralogs belonging to each fish species are labelled with the same color. 17](#_Toc67649487)

[Fig. 5. Changes in the number of subfamily β (A) and ε (B) ORs during the evolution of 28 fish species inferred from intact genes. 18](#_Toc67649488)

# Table 1 Pacific BioSciences raw read statistics.

| N50 (bp) | 12,735 |
| --- | --- |
| Mean length (bp) | 10,062 |
| Max length (bp) | 53,359 |
| Reads number | 8,268,554 |
| Clean base (Gb) | 83.20 |
| Depth (X) | 74.29 |

# Table 2 Statistics of genome assembly.

|  | **Scaffold Length (bp)** | **Scaffold Number** | **Contig Length (bp)** | **Contig Number** |
| --- | --- | --- | --- | --- |
| N50 | 3,154,243 | 95 (10.94%) | 2,397,079 | 112 (7.36%) |
| N60 | 2,312,875 | 136 (15.67%) | 1,754,877 | 166 (10.91%) |
| N70 | 1,732,860 | 191 (22.00%) | 1,195,390 | 243 (15.97%) |
| N80 | 1,137,094 | 270 (31.11%) | 748,399 | 357 (23.46%) |
| N90 | 671,479 | 393 (45.28%) | 355,306 | 570 (37.45%) |
| Longest | 17,436,821 |  | 17,436,821 |  |
| Total Size | 1,109,245,993 |  | 1,107,959,078 |  |
| Total Number |  | 868 |  | 1,522 |

# Table 3 Genome assembly completeness using BUSCO.

| **Categories** | **Number** | **Percent (%)** |
| --- | --- | --- |
| Complete BUSCOs (C) | 4404 | 96.1 |
| Complete and single-copy BUSCOs (S) | 4212 | 91.9 |
| Complete and duplicated BUSCOs (D) | 192 | 4.2 |
| Fragmented BUSCOs (F) | 59 | 1.3 |
| Missing BUSCOs (M) | 121 | 2.6 |
| Total BUSCO groups searched | 4584 | 100 |

Note: BUSCO version is: 2.0; The lineage dataset is: actinopterygii_odb9 (Creation date: 2016-02-13, number of species: 20, number of BUSCOs: 4,584).

# Table 4 Scaffolding statistics based on linkage map data for each chromosome.

| **Chromosome** | **No. of markers** | **No. of scaffolds** | **Chromosome**  **size (Mb)** |
| --- | --- | --- | --- |
| 1 | 784 | 91 | 75.86 |
| 2 | 768 | 42 | 42.12 |
| 3 | 805 | 27 | 62.69 |
| 4 | 653 | 21 | 62.79 |
| 5 | 838 | 26 | 40.95 |
| 6 | 604 | 21 | 56.49 |
| 7 | 552 | 26 | 36.45 |
| 8 | 645 | 11 | 44.27 |
| 9 | 609 | 26 | 36.69 |
| 10 | 563 | 13 | 44.06 |
| 11 | 693 | 25 | 46.57 |
| 12 | 591 | 22 | 58.60 |
| 13 | 555 | 17 | 40.86 |
| 14 | 512 | 56 | 52.87 |
| 15 | 584 | 28 | 38.51 |
| 16 | 603 | 28 | 42.27 |
| 17 | 521 | 33 | 48.95 |
| 18 | 518 | 27 | 40.11 |
| 19 | 448 | 15 | 33.88 |
| 20 | 394 | 15 | 40.96 |
| 21 | 379 | 23 | 35.21 |
| 22 | 481 | 22 | 33.38 |
| 23 | 240 | 11 | 35.37 |
| 24 | 313 | 24 | 28.48 |
| Total | 13,653 | 650 | 1078.39 |

# Table 5 28 fish genome information.

|  | Species | Accession numbers |
| --- | --- | --- |
| 1 | *M. amblycephala* | This study |
| 2 | *D. rerio* | GCA_000002035.4 |
| 3 | *C. idellus* | http://www.ncgr.ac.cn/grasscarp/ |
| 4 | *C. carpio* | GCA_000951615.2 |
| 5 | *I. punctatus* | GCA_001660625.1 |
| 6 | *S. meridionalis* | PRJNA427243 |
| 7 | *M. albus* | GCA_001952655.1 |
| 8 | *L. chalumnae* | GCA_000225785.1 |
| 9 | *L. oculatus* | GCA_000242695.1 |
| 10 | *C. harengus* | GCA_900700415.1 |
| 11 | *S. grahami* | GCA_001515645.1 |
| 12 | *P. hyalocraniu* | ftp://parrot.genomics.cn/gigadb/pub/10.5524/100001_101000/100262/ |
| 13 | *G. morhua* | GCA_000231765.1 |
| 14 | *O. latipes* | GCA_002234675.1 |
| 15 | *X. maculatus* | GCA_000241075.1 |
| 16 | *S. salar* | GCA_000233375.4 |
| 17 | *H. comes* | GCA_001891065.1 |
| 18 | *G. aculeatus* | GCA_006229165.1 |
| 19 | *L. crocea* | GCA_000972845.2 |
| 20 | *L. calcarifer* | GCA_001640805.1 |
| 21 | *O. niloticus* | GCA_001858045.3 |
| 22 | *C. semilaevis* | GCA_000523025.1 |
| 23 | *T. rubripes* | GCA_000180615.2 |
| 24 | *M. mola* | GCA_001698575.1 |
| 25 | *A. anguilla* | GCA_000695075.1 |
| 26 | *A. mexicanus* | GCA_000372685.1 |
| 27 | *P. olivaceus* | GCA_001904815.2 |
| 28 | *S. formosus* | GCA_900964775.1 |

# Table 6 Comparison of intact OR genes in 28 fish and other vertebrates.

| **Species** | **Water** | | | |  | **Air** | |  | **Air/Water** |  | **Non-OR** | |  | **Total** |
| --- | --- | --- | --- | --- | --- | --- | --- | --- | --- | --- | --- | --- | --- | --- |
|  | **Delta**  **(δ)** | **Epsilon**  **(ε)** | **Zeta**  **(ζ)** | **Eta**  **(η)** |  | **Alpha**  **(α)** | **Gamma**  **(γ)** |  | **Beta**  **(β)** |  | **Kappa**  **(κ)** | **Thet**  **(θ)** |  |  |
| *G. aculeatus* | 86 | 4 | 18 | 11 |  | 0 | 3 |  | 1 |  | 1 | 1 |  | 125 |
| *L. crocea* | 57 | 3 | 10 | 46 |  | 0 | 0 |  | 2 |  | 1 | 1 |  | 120 |
| *M. mola* | 11 | 1 | 1 | 6 |  | 0 | 0 |  | 0 |  | 1 | 0 |  | 20 |
| *T. rubripes* | 39 | 2 | 4 | 13 |  | 0 | 0 |  | 1 |  | 1 | 1 |  | 61 |
| *M. albus* | 93 | 2 | 65 | 5 |  | 0 | 0 |  | 1 |  | 1 | 1 |  | 168 |
| *P. olivaceus* | 26 | 2 | 5 | 6 |  | 0 | 0 |  | 1 |  | 1 | 1 |  | 42 |
| *C. semilaevis* | 59 | 3 | 9 | 16 |  | 0 | 0 |  | 1 |  | 1 | 1 |  | 90 |
| *L. calcarifer* | 165 | 5 | 24 | 76 |  | 0 | 0 |  | 7 |  | 1 | 1 |  | 279 |
| *O. niloticus* | 97 | 4 | 29 | 34 |  | 0 | 1 |  | 5 |  | 1 | 2 |  | 173 |
| *X. maculatus* | 38 | 4 | 4 | 12 |  | 0 | 0 |  | 1 |  | 1 | 1 |  | 61 |
| *O. latipes* | 29 | 2 | 9 | 18 |  | 0 | 0 |  | 2 |  | 1 | 0 |  | 61 |
| *H. comes* | 18 | 1 | 1 | 3 |  | 0 | 0 |  | 0 |  | 0 | 1 |  | 24 |
| *G. morhua* | 55 | 2 | 13 | 11 |  | 0 | 0 |  | 1 |  | 0 | 1 |  | 83 |
| *S. salar* | 40 | 6 | 13 | 10 |  | 0 | 2 |  | 2 |  | 2 | 2 |  | 77 |
| *P. hyalocraniu* | 67 | 6 | 3 | 23 |  | 0 | 1 |  | 2 |  | 0 | 2 |  | 104 |
| *C. harengus* | 57 | 5 | 6 | 21 |  | 0 | 5 |  | 0 |  | 1 | 1 |  | 96 |
| *S. meridionalis* | 31 | 16 | 15 | 23 |  | 0 | 0 |  | 11 |  | 1 | 1 |  | 98 |
| *I. punctatus* | 31 | 11 | 19 | 13 |  | 0 | 0 |  | 9 |  | 1 | 1 |  | 85 |
| *A. mexicanus* | 32 | 9 | 20 | 26 |  | 0 | 5 |  | 28 |  | 1 | 1 |  | 122 |
| *S. grahami* | 50 | 11 | 28 | 21 |  | 0 | 1 |  | 5 |  | 2 | 2 |  | 120 |
| *C. carpio* | 100 | 12 | 46 | 38 |  | 0 | 1 |  | 15 |  | 2 | 3 |  | 217 |
| *C. idellus* | 55 | 11 | 25 | 18 |  | 0 | 1 |  | 14 |  | 1 | 1 |  | 126 |
| *M. amblycephala* | 113 | 13 | 43 | 29 |  | 0 | 3 |  | 20 |  | 1 | 1 |  | 223 |
| *D. rerio* | 64 | 13 | 35 | 37 |  | 0 | 1 |  | 7 |  | 1 | 1 |  | 159 |
| *S. formosus* | 35 | 8 | 10 | 16 |  | 0 | 1 |  | 9 |  | 1 | 1 |  | 81 |
| *A. anguilla* | 67 | 1 | 25 | 9 |  | 0 | 2 |  | 1 |  | 1 | 2 |  | 108 |
| *L. oculatus* | 57 | 4 | 30 | 19 |  | 3 | 34 |  | 18 |  | 1 | 2 |  | 168 |
| *L. chalumnae* | 69 | 0 | 47 | 16 |  | 11 | 15 |  | 0 |  | 3 | 1 |  | 162 |
| Western clawed frog | 27 | 13 | 0 | 10 |  | 8 | 752 |  | 14 |  | 3 | 1 |  | 828 |
| Lizard | 0 | 0 | 0 | 0 |  | 1 | 111 |  | 0 |  | 1 | 1 |  | 114 |
| Chicken | 0 | 0 | 0 | 0 |  | 9 | 202 |  | 0 |  | 1 | 0 |  | 212 |
| Platypus | 0 | 0 | 0 | 0 |  | 31 | 234 |  | 0 |  | 1 | 0 |  | 266 |
| Cow | 0 | 0 | 0 | 0 |  | 140 | 828 |  | 2 |  | 0 | 1 |  | 971 |
| Chimpanzee | 0 | 0 | 0 | 0 |  | 64 | 316 |  | 0 |  | 0 | 0 |  | 380 |
| Human | 0 | 0 | 0 | 0 |  | 58 | 329 |  | 0 |  | 1 | 0 |  | 388 |

The number of OR genes of tetrapods labeled green are from Niimura (2009).

# Table 7 Results from aBSREL analysis applied to Fig. 5

| Genes | Model^a^ | lnL | AICc^b^ | ω rate classes^c^ | Number of branches | % of branches | Branches under selection |
| --- | --- | --- | --- | --- | --- | --- | --- |
| Beta | MG94 | -39065.85 | 79466.02 | 1 | 233 | 73 | 4 (Bonferroni-Holm corrected *P*-value <0.05) |
|  | Full model | -38454.05 | 78606.5 | 2 | 87 | 27 |  |
| Epsilon | MG94 | -37057.32 | 75423.62 | 1 | 215 | 69 | 3 (Bonferroni-Holm corrected *P*-value <0.05) |
|  | Full model | -36388.18 | 74498.13 | 2 | 98 | 31 |  |

^a^ Best-fit model

^b^ Akaike Information Criteria

^c^ Omega rate classes established by full model.

Table 8 Sampling information for RNA-Seq and qRT-PCR in this study. OB, olfactory bulb; B, brain, OE, olfactory epithelium.

|  | Fish species | Fish number | Library number | Sex | Age | Habitat | Tissue | Accession number |
| --- | --- | --- | --- | --- | --- | --- | --- | --- |
| RNA-Seq | *M. amblycephala* | 3 | 3 | Male | Adult | Freshwater | OB | This study |
|  | *M. amblycephala* | 3 | 3 | Male | Adult | Freshwater | B |  |
|  | *M. amblycephala* | 3 | 3 | Male | Adult | Freshwater | OE |  |
|  | *D. rerio* | 3 | 3 | Male | Adult | Freshwater | OE | ERR375744, ERR375745, ERR375746, ERR375747, ERR375748, ERR375749 |
|  | *A. anguilla* | 9 | 3 | Male | Immature | Freshwater | OE | SRR1564732, SRR1564733, SRR1564734 |
|  | *A. anguilla* | 9 | 3 | Male | Immature | Seawater | OE | SRR1574814, SRR1574939  SRR1574942 |
|  | *A. anguilla* | 9 | 3 | Male | Mature | Seawater | OE | SRR1574946, SRR1574947  SRR1574950 |
| qRT-PCR | *M. amblycephala* | 9 | 3 | Male | Adult | Freshwater | M | This study |
|  |  |  |  |  |  |  | OE |  |
|  |  |  |  |  |  |  | OB |  |
|  |  |  |  |  |  |  | B |  |
|  |  | 9 | 3 | Female | Adult | Freshwater | M |  |
|  |  |  |  |  |  |  | OE |  |
|  |  |  |  |  |  |  | OB |  |
|  |  |  |  |  |  |  | B |  |

Table 9 Primers of representative β and ε OR genes in *M. amblycephala* for qRT-PCR. F, forward; R, reverse.

| **Gene name** | **Sequence (5'—3')** | **Product size (bp)** |
| --- | --- | --- |
| *M.a-*β-actin-F | ACCCACACCGTGCCCATCTA | 104 |
| *M.a-*β-actin-R | CGGACAATTTCTCTTTCGGCTG |  |
| *M.a-*β2-F | TTGTGGATGGCACTGGATCG | 204 |
| *M.a-*β2-R | TGCCATGTGCTCACAAAAACA |  |
| *M.a-*β9-F | GCTTTTGTGGATGGCACTGG | 287 |
| *M.a-*β9-R | AGTCTGCAGTTGGGATCAGA |  |
| *M.a-*β10-F | GGCTTTAGATCGCTTTGCGG | 215 |
| *M.a-*β10-R | CACAGGCCAGGCTTACAAGA |  |
| *M.a-*β11-F | CCGTAACTTGCTTTGTGTTGT | 246 |
| *M.a-*β11-R | GACTCACCAGAAGGAGAGCG |  |
| *M.a*-ε6-F | ACAGAAGAACTGGACGACGA | 162 |
| *M.a*-ε6-R | AGGCTCCGTGAAGTGACAAA |  |
| *M.a*-ε7-F | GGGAAACATCAGCTTTGTAAGGG | 126 |
| *M.a*-ε7-R | ACAGCATTTCCCACCAGTGT |  |
| *M.a*-ε10-F | TTGGCATGTGCTGCGTTTTG | 155 |
| *M.a*-ε10-R | TGCCTTACATCTCCACAAGACA |  |
| *M.a*-ε13-F | GGCAGCAATGACGGAACCTA | 148 |
| *M.a*-ε13-R | TCCCAGTAGCAAGAAGAAGGT |  |

**Figure 1**

**
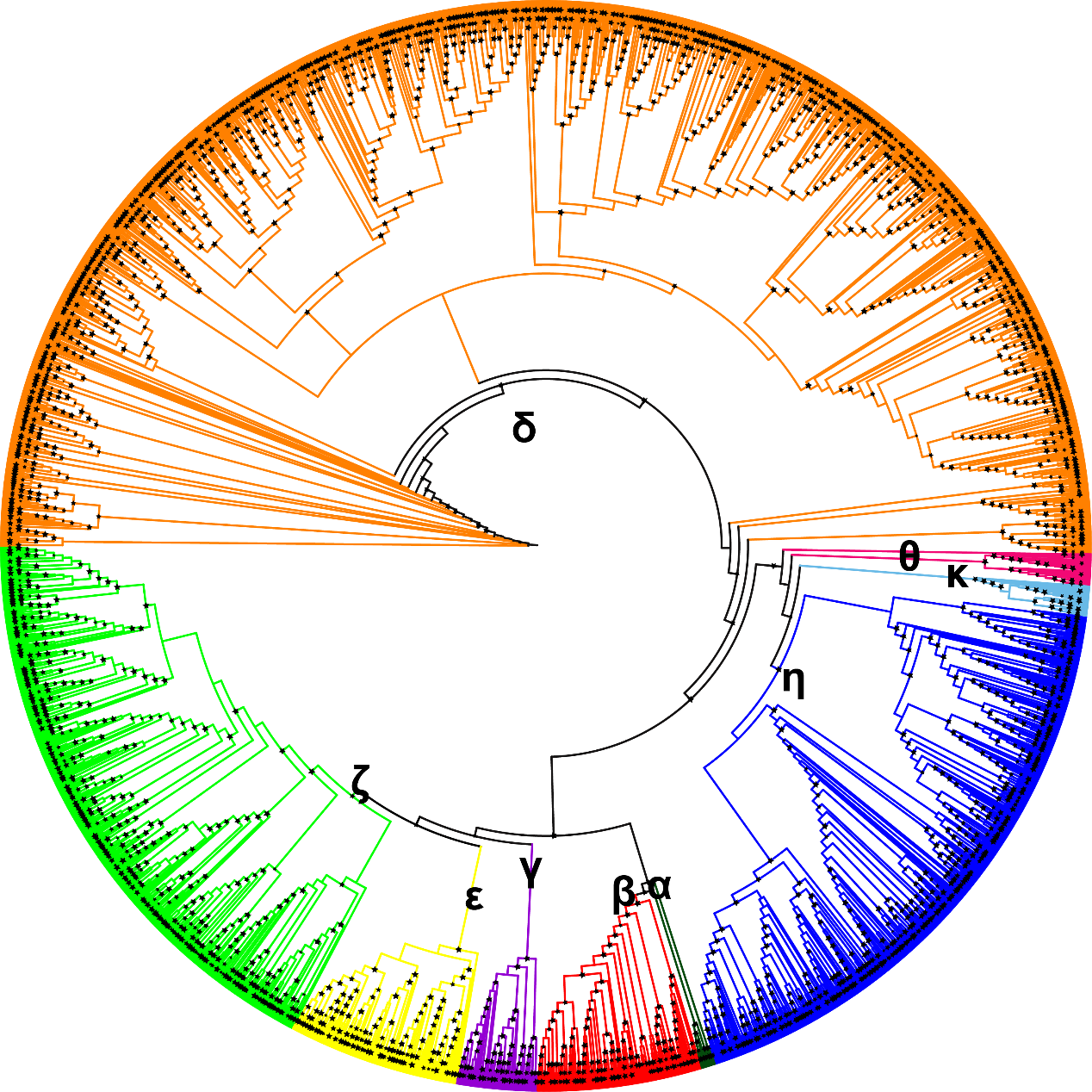
**

Fig. 1. ML tree of the intact OR gene families identified across 28 fish genomes (n = 3,253). All major clades had bootstrap values greater than 70% (1,000 replicates).

**Figure 2**


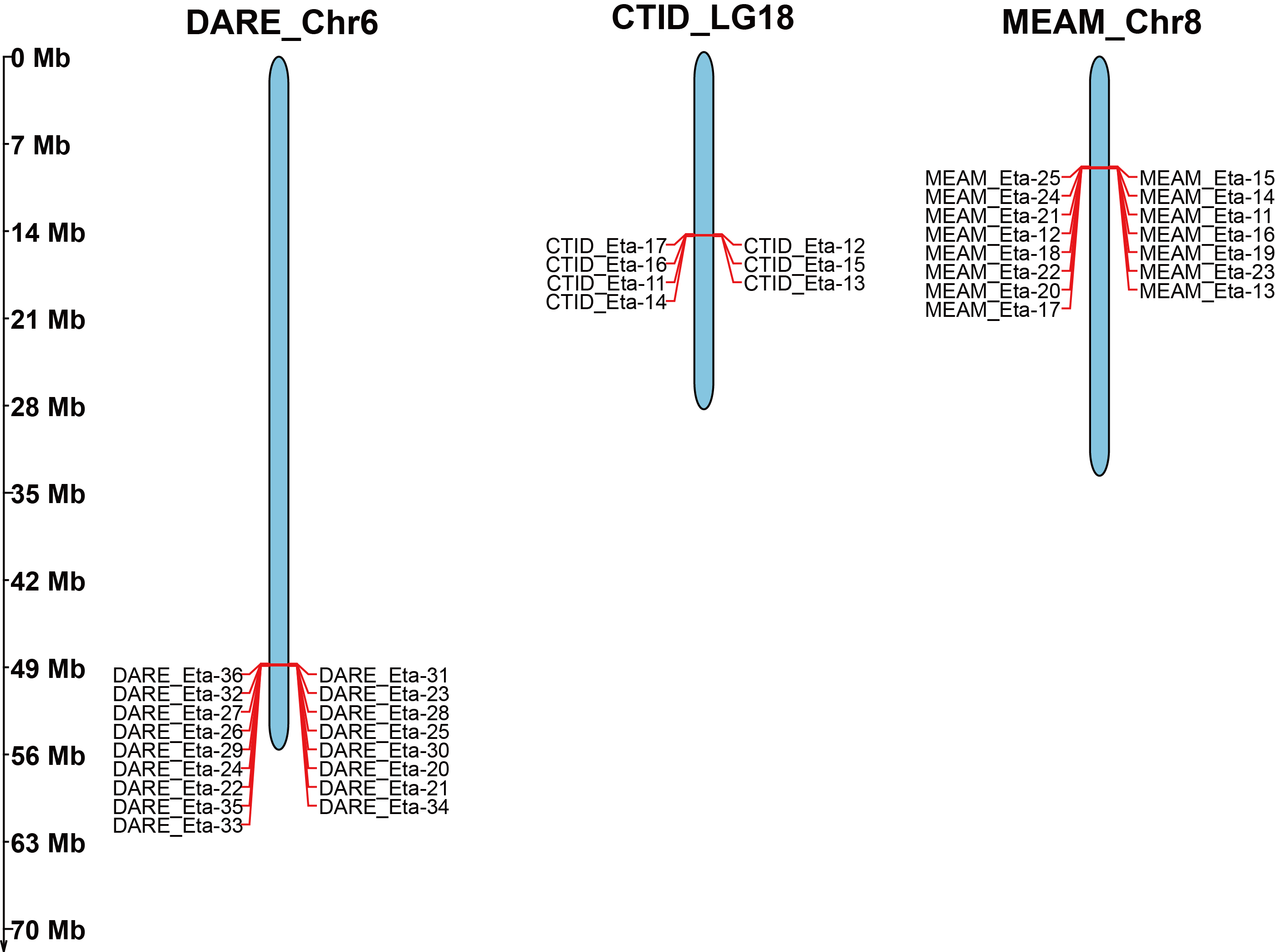


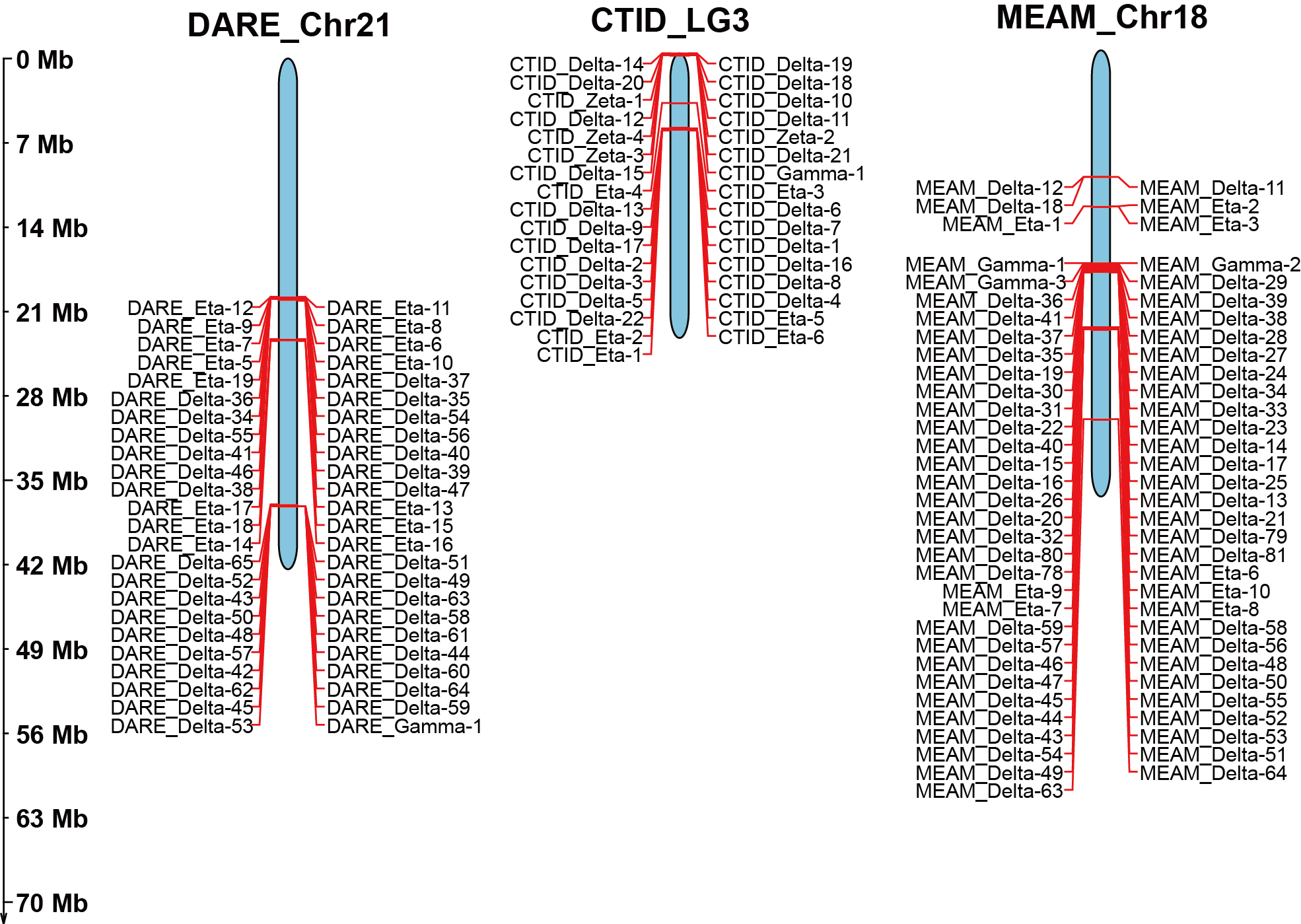


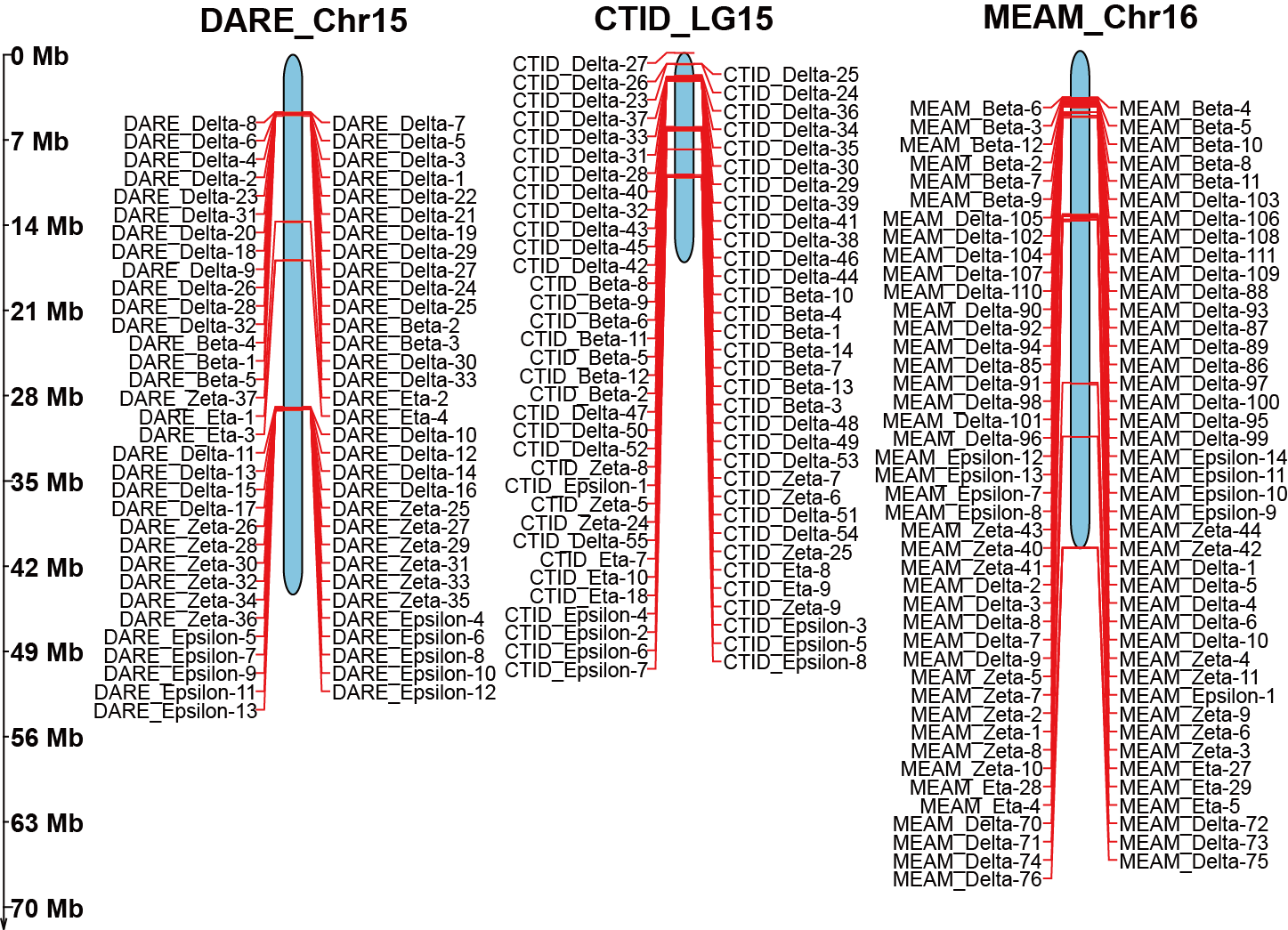


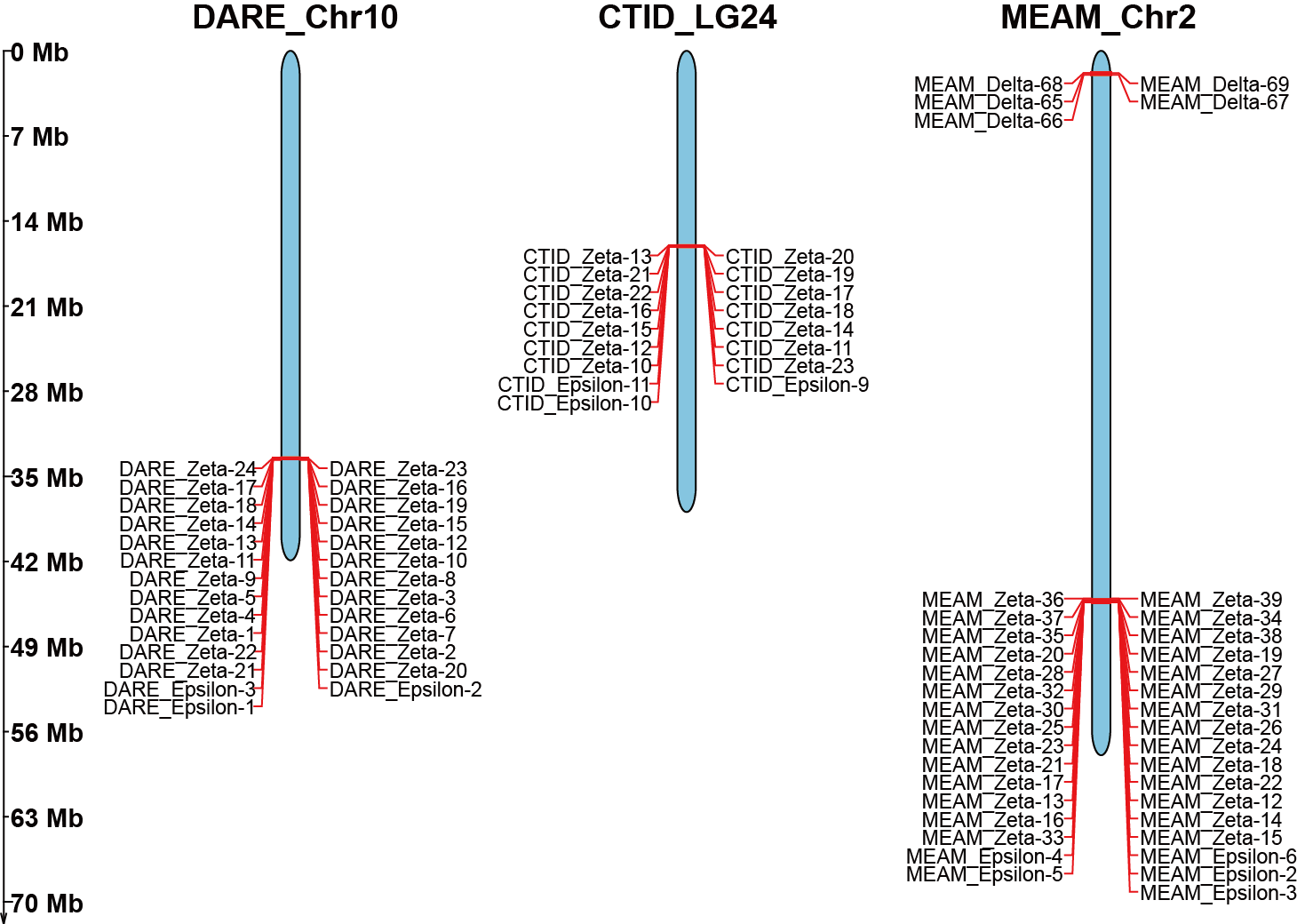


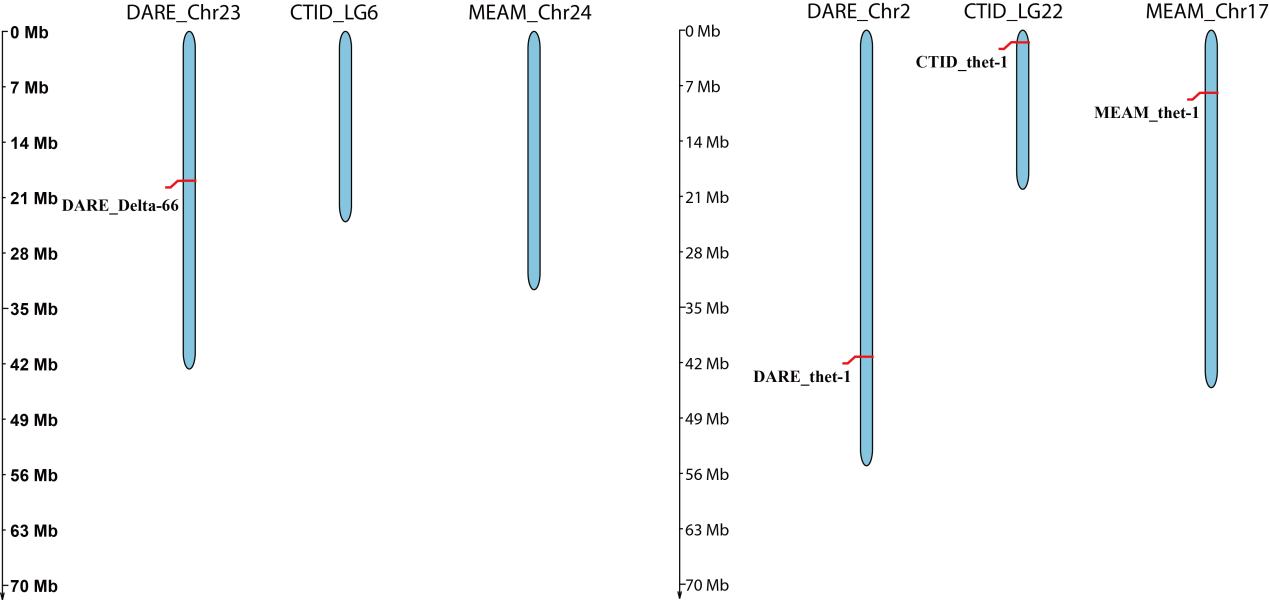

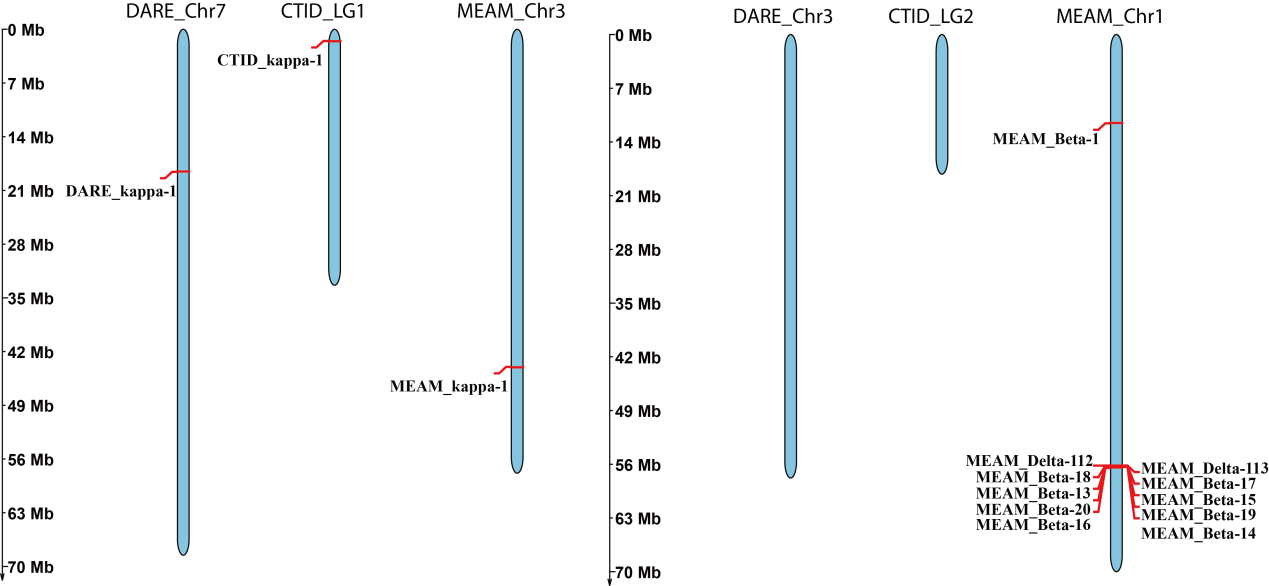


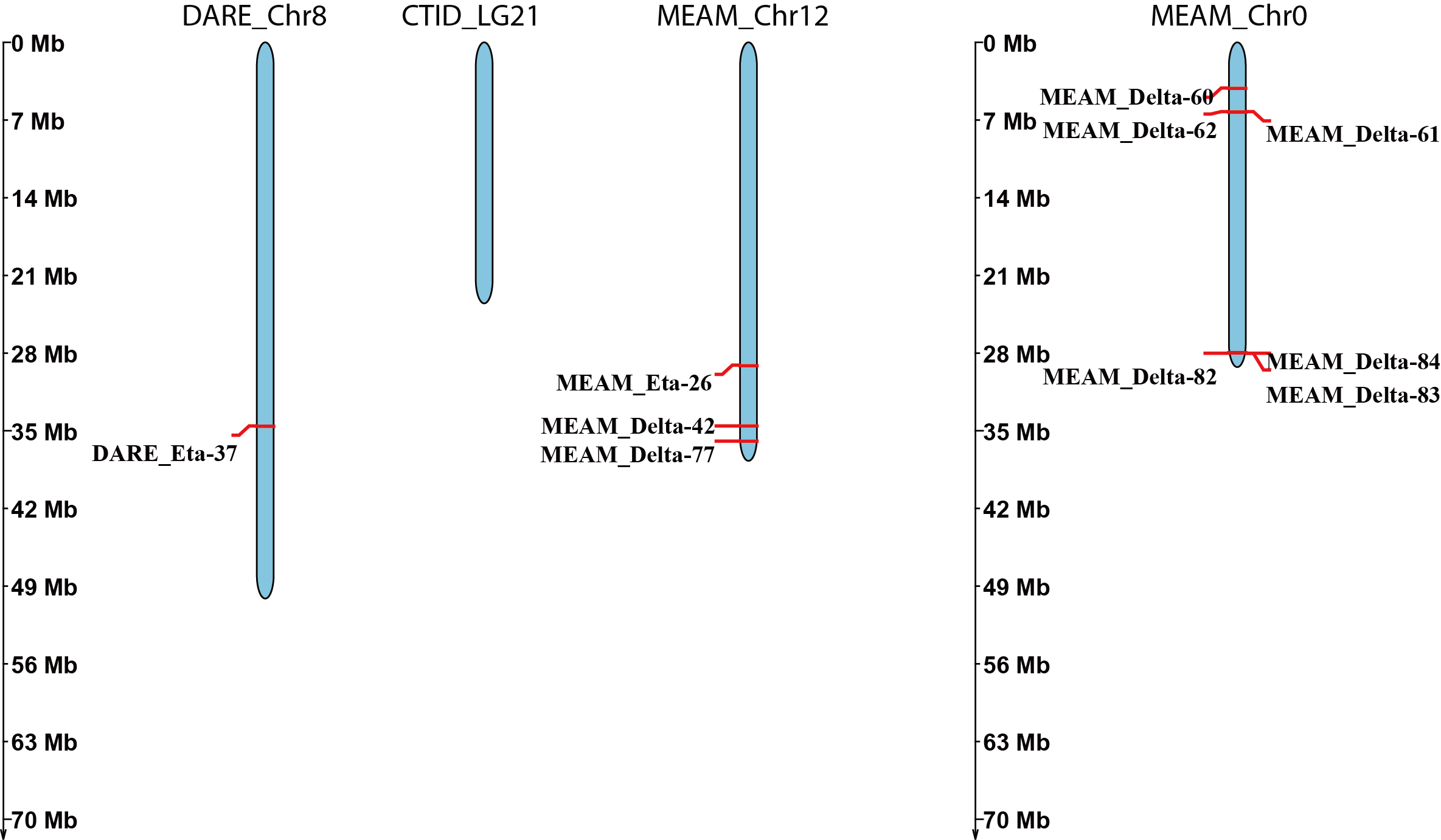


Fig. 2. Physical maps of intact OR genes in several genomic clusters among *D. rerio*, *C. idellus* and *M. amblycephala*. The position of each OR gene is represented by a horizontal bar.

**Figure 3**

**
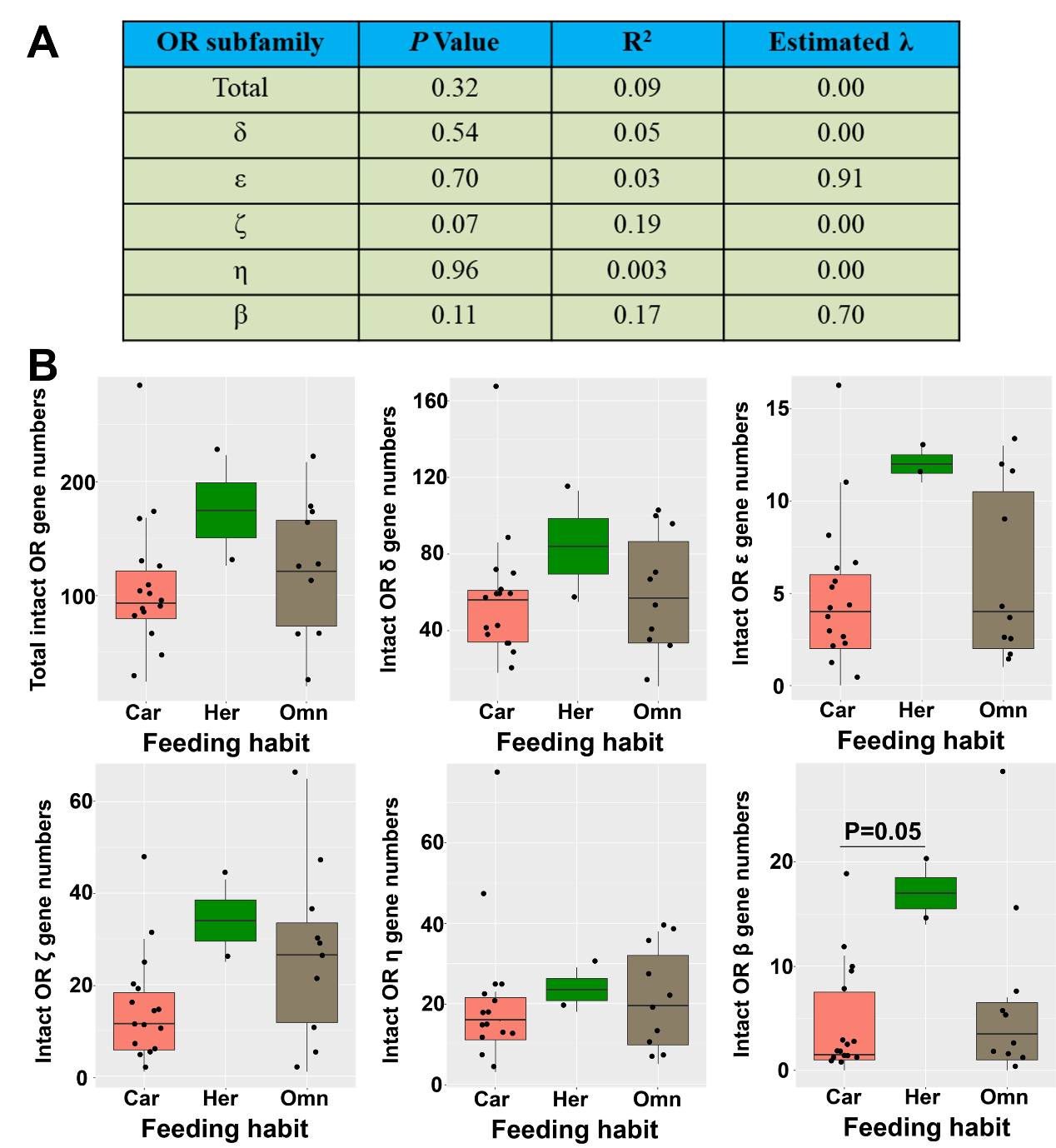
**

Fig. 3. OR genes subfamilies were compared among fish with different feeding habits. **(A)** Phylogenetic generalized least squares (PGLS) regression analysis for number of intact OR genes in fish species (n=28) versus feeding habits**.** (B) Box plot of intact OR gene numbers among groups of different feeding habits. One-way ANOVA analysis and Tukey HSD test were conducted to determine statistical difference among groups. Car, carnivorous; Omn, omnivorous; Her, herbivorous.

**Figure 4**

**
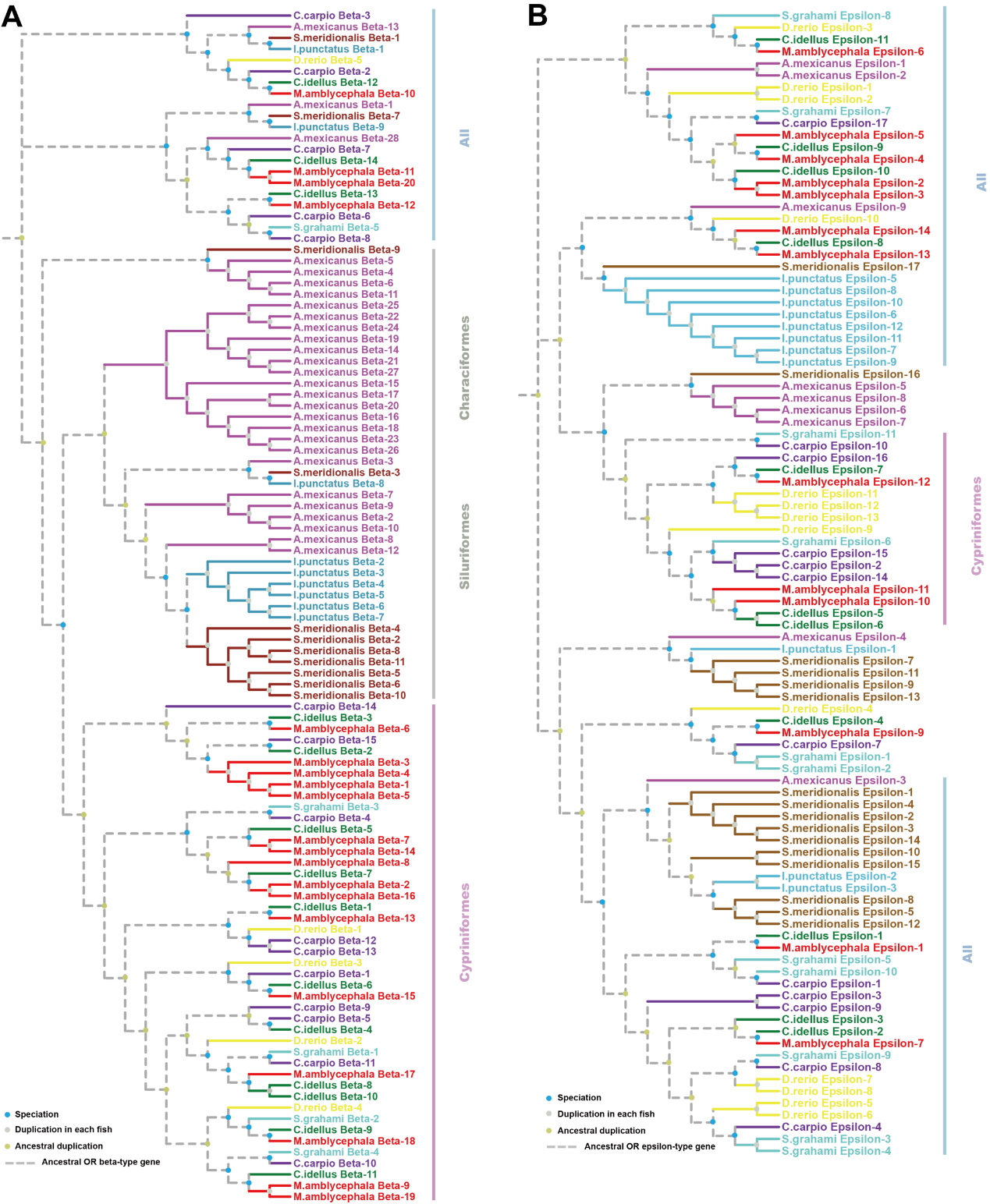
**

# Fig. 4. Diversification of intact subfamily β (A) and ε (B) OR genes in the Otophysa lineage. Paralogs belonging to each fish species are labelled with the same color.

**Figure 5**


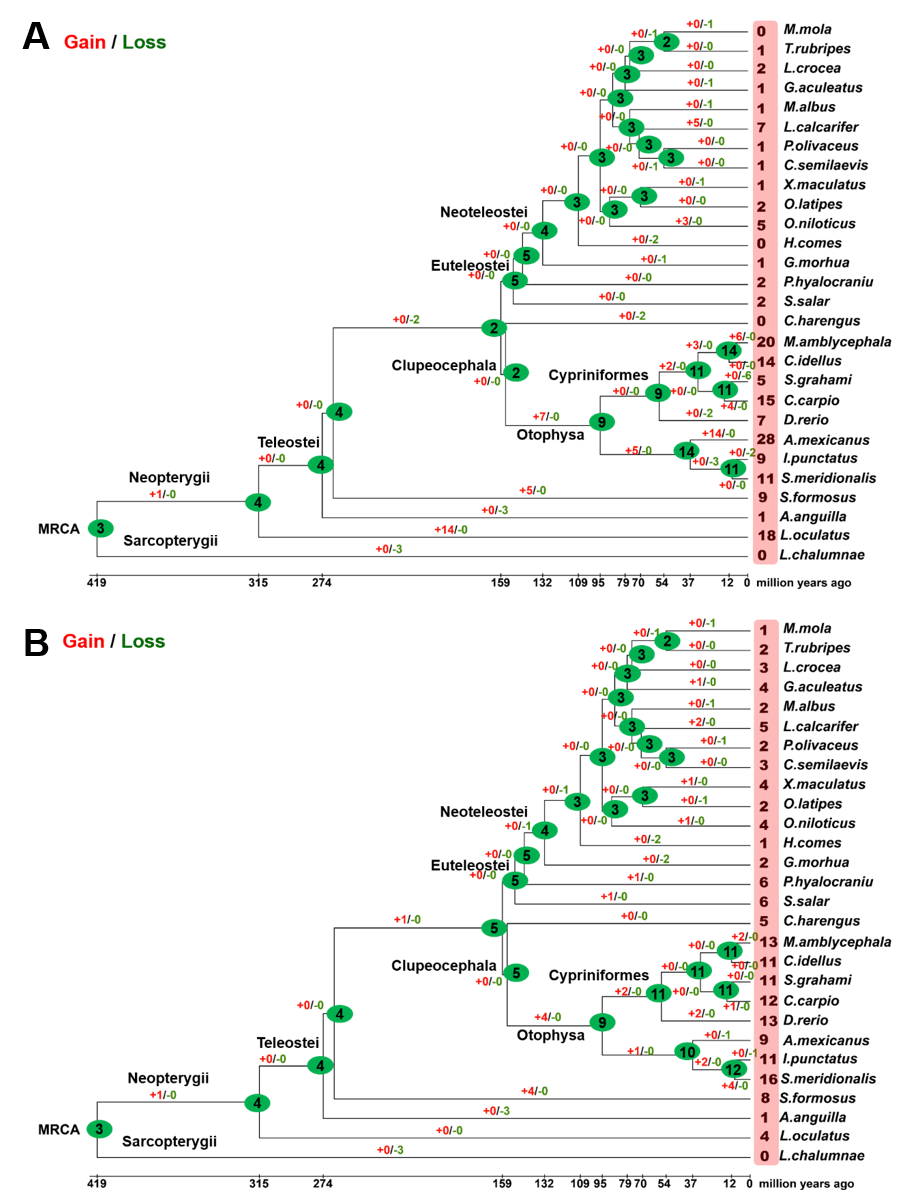


# Fig. 5. Changes in the number of subfamily β (A) and ε (B) ORs during the evolution of 28 fish species inferred from intact genes.
